# New Molecular Insights on Gabapentin

**DOI:** 10.1021/acsphyschemau.4c00108

**Published:** 2025-06-03

**Authors:** Sofía Municio, Sergio Mato, José L. Alonso, Elena R. Alonso, Iker León

**Affiliations:** Grupo de Espectrocopía Molecular (GEM), Edificio Quifima, Laboratorios de Espectroscopia y Bioespectroscopia, Unidad Asociada CSIC, Parque Científico UVa, 16782 Universidad de Valladolid, Valladolid 47011, Spain

**Keywords:** gabapentin, FTMW spectroscopy, noncovalent interactions, drugs, GABA

## Abstract

Neutral gabapentin has been vaporized by laser ablation and supersonically expanded to record its rotational spectrum using Fourier transform microwave spectroscopy. We report the detection of five stable conformers, which differ in the intramolecular interactions between the different functional groups (OH, C=O, and NH). Two configurations, *axial* and *equatorial*, are possible depending on the chair form of the cyclohexane ring, and both forms are detected, with the latter being predominant. The conformational landscape of gabapentin is compared with that of GABA, and significant differences are observed. One of the most meaningful results of such a comparison is that the relationship between the intramolecular interactions and the relative abundance within each type is reversed from GABA to gabapentin. It could explain the distinction in the mechanism of action of GABA and gabapentin, despite being structurally similar.

## Graphical abstract

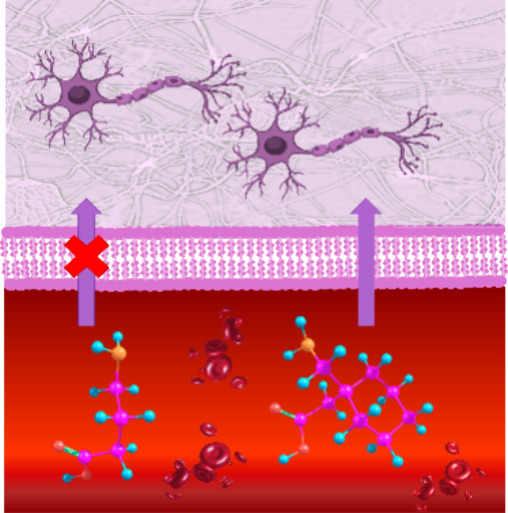

## Introduction

Gabapentin is an anticonvulsant drug used as a medication to treat epilepsy and manage neuropathic pain or anxiety disorder.^1^ Gabapentin shares a remarkable similarity in structure (see Figure 1a), function, and clinical uses with the neurotransmitter GABA. For example, it reduces uncontrolled and repetitive neuronal activity,^2^ asynchronous neuronal discharge, or nervous system disorders.^3^

**Figure 1:**
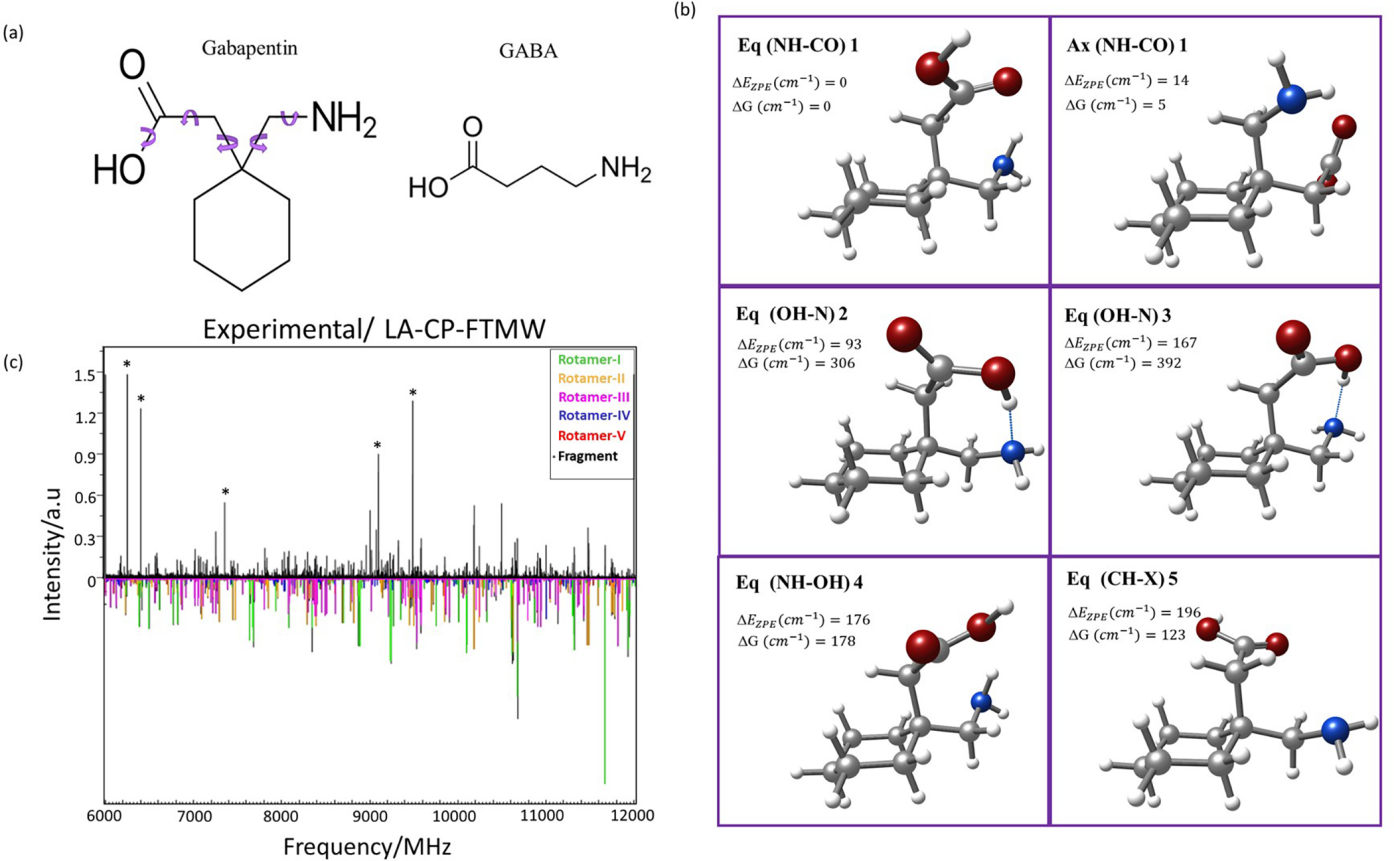
(a) Structures of gabapentin and GABA. The chemical structure of gabapentin is derived by adding a cyclohexyl group to the backbone of GABA. The arrows indicate the different torsional degrees of freedom that give rise to different structural conformers of gabapentin. (b) Summary of the six most stable conformers of the gabapentin molecule. The upper left shows the energy difference considering the zero-point energy correction (Δ*E*_ZPE_), as well as the entropic difference at room temperature and 1 bar (Δ*G*). The conformers are numbered as described in the text. For easier tracking of the structures in the figure, the intramolecular interaction of each conformer is shown in parentheses. (c) Broadband LA-CP-FTMW rotational spectrum of gabapentin in the 6000–12000 MHz range (see also Figure S02), together with the simulated rotational spectra for the five conformers detected (each in a different color).

The blood-brain barrier (BBB), while essential for protecting the brain from harmful substances, can also impede the delivery of potentially beneficial medications. It was initially thought that GABA is unable to cross the BBB due to its impermeability.^4–8^ By developing gabapentin as a GABA analogue with an enhanced ability to cross this barrier, the researchers at Parke-Davis sought to create a more effective therapeutic agent for neurological conditions. However, despite its structural similarity to GABA, gabapentin’s mechanism of action appears to be different from that of GABA and does not directly interact with GABA receptors. Instead, its mechanism of action is believed to involve modulation of voltage-gated calcium channels, mainly through binding to the α2δ subunit of a voltage-dependent Ca^2+^ channel.^4,9–13^

In recent years, there has been intense interest in determining the structure of gabapentin. Gabapentin can exist in two distinct conformations corresponding to the two interconvertible chair forms of the cyclohexane ring. This chair form configuration is essential as it can have drastic implications for interaction at a receptor site. Therefore, several studies have been devoted to obtaining this structure in condensed phases. In the solid state, four polymorphs have been reported so far.^14,15^ In most of the characterized forms of gabapentin, the molecule crystallizes as a zwitterion, with the aminomethyl group mainly occupying the *axial* position.^15^ Interestingly, for the hydrochloride hemihydrate of gabapentin, there is an inversion of the cyclohexane ring, and the aminomethyl group is in the *equatorial* position due to the different packing forces.^16^ In solution, NMR studies establish a rapid conformational exchange between both forms at room temperature, while at low temperatures, which permit conformational freezing, the most stable conformer has the aminomethyl group in the *equatorial* position.^16^ In fact, low temperature ^1^H NMR techniques suggest that the probable binding conformation of gabapentin is with the aminomethyl moiety in the *equatorial* frame in relation to the cyclohexane ring.^17^ However, no study exists in the gas phase and thus its isolated structure is unknown. Because the intrinsic conformational choices of gabapentin can be revealed when studied in isolation conditions, in this paper, we present the results obtained from gabapentin using rotational spectroscopy. Additionally, the conformational panorama will be contrasted against that of GABA, to evaluate if any conformational difference could justify their completely different binding sites.

## Materials and Methods

### Experimental Methods

We used a commercial sample of gabapentin without any further purification. The preparation of the solid rod was carried out by pressurization of the compound mixed with a small amount of commercial binder (Peoval 33) and then it was placed in the ablation nozzle. A picosecond Nd:YAG laser (20 mJ per pulse, 20 ps pulse width) was used as a vaporization tool. Products of the laser ablation were supersonically expanded utilizing the flow of carrier gas (Ne, 8 bar) and characterized by both chirped-pulse Fourier transform microwave spectroscopy (LA-CP-FTMW) and molecular beam Fourier transform microwave spectroscopy (LA-MB-FTMW), using a recent constructed instrument^18,19^ dedicated to maximize its performance from 6 to 12 GHz. The LA-CP-FTMW spectrometer enables the acquisition of a broadband (several GHz) rotational spectrum to identify all the conformers, while the LA-MB-FTMW spectrometer is ideal for providing the high-resolution necessary to analyze the hyperfine structure due to the presence of several ^14^N nuclei in the molecule. In the LA-MB-FTMW spectrometer all the transitions appeared as Doppler doublets due to the parallel configuration of the molecular beam and the microwave radiation. In this case, the resonance frequency was determined as the arithmetic mean of the two Doppler components.

### Computational Methods

The five hindered rotations around the single bonds and the change in the conformation of the six-carbon ring, wheather a chair, boat or intermediate conformation, generate a plethora of conformational species. Therefore, the conformational space of gabapentin was first explored using fast molecular mechanics methods. *MMFFs* ^20^ and AMBER^21^ force fields were both used.

Geometry optimizations of gabapentin were done using Gaussian suite programs.^22^ The selected model for the primary investigation was an advanced DFT method based on a double-hybrid density functional (B2PLYPD) with long-range dispersion corrections,^23^ a mixed method between Mo̷ller-Plesset (MP2) and DFT methods, with the Pople’s 6-311++G­(d,p) basis set.^24^ In order to contrast the results with cheaper methodologies, MP2 ^25^ and B3LYP^26–28^ were also conducted using the same basis set. Frequency calculations were also computed to ensure that the optimized geometries are true minima and to calculate the Gibbs free energies.

## Results and Discussion

### Conformational Landscape

Initially, a deep search for the most stable conformations of neutral gabapentin was carried out. At first instance, several molecular structures were screened using molecular mechanics calculations. 98 structures were generated within an energetic range of 2500 cm^–1^ (30 kJ/mol). These structures were subsequently optimized using Gaussian suite programs^22^ through quantum mechanics methods, using B3LYP,^26–28^ MP2 ^25^ and the double hybrid functional B2PLYP,^23^ all with the 6-311++G­(d,p) basis set.^24^ For B3LYP and B2PLYP, Grimme dispersion and Becke Jonhson corrections were included.^29^ A total of 37 structures were obtained below 1000 cm^–1^ (12 kJ/mol). All the structures are collected in Figure S01 of the Supporting Information, while Tables S01 to S03 collect the derived spectroscopic parameters at different calculation levels, and Table S04 shows the Cartesian coordinates at B2PLYP-GD3BJ/6-311++G­(d,p). The most stable structures in a 200 cm^–1^ (2.4 kJ/mol) energy window relative to the global minimum are shown in Figure 1b, and the rotational constants, nuclear quadrupole coupling constants, and electric dipole moment components are collected in Table 1. The conformers have been labeled based on the following considerations: *ax* and *eq* terms have been used to account for the *axial* and *equatorial* disposition of the aminomethyl group to match the labeling of the published literature; next, the number indicates the energetic ordering of each conformation at B2PLYP-GD3BJ/6-311++G­(d,p), which gives best results for this molecular system.

**Table 1:** Experimental Spectroscopic Parameters Obtained for the Detected Rotamers 1–5 of Gabapentin Compared with Those Calculated Using B2PLYP/6-311++G­(d,p) for the Six Lowest Energy Conformers

	experimental					B2PLYP/6-311++G(d,p)					
	rotamer 1^h^	rotamer 2	rotamer 3	rotamer 4	rotamer 5	*eq1*	*ax1*	*eq2*	*eq3*	*eq4*	*eq5*
*A* ^a^	1272.682(37)^g^	1486.29069(309)	1218.0263(86)	1361.8952(108)	1311.2210(65)	1277	1489	1220	1361	1316	1131
*B*	757.6391(280)	652.8589(43)	790.6139(80)	722.2271(75)	736.2311(210)	757	652	790	723	736	820
*C*	572.89083(246)	546.14537(44)	610.46391(75)	570.33260(49)	570.4553(130)	573	546	610	571	571	579
|μ_*a*_|	observed	observed	observed	observed	not observed	0.9	0.8	2.3	3.3	0.5	1.6
|μ_*b*_|	observed	observed	observed	observed	observed	0.7	1.0	6.4	5.6	1.4	0.3
|μ_*c*_|	observed	not observed	not observed	not observed	not observed	0.8	0.5	0.1	0.0	0.5	0.4
χ_*aa*_	–0.003(79)	1.073(26)	0.891(85)	–0.001(48)		–0.007	1.022	1.037	–0.072	–0.253	1.855
χ_*bb*_	2.014(62)	2.356(20)	–2.296(65)	–0.392(35)		2.269	2.690	–2.562	–0.602	2.135	1.095
χ_*cc*_	–2.010(52)	–3.429(20)	1.405(65)	0.399(35)		–2.261	–3.712	1.524	0.673	–1.882	–2.950
σ^b^	3	2.5	3.4	2.4	100						
N^c^	12	12	14	11	11						
Δ*E* ^d^						52	70	0	23	262	327
Δ*E*_ZPE_ ^e^						0	14	93	167	176	197
Δ*G* ^f^						0	5	306	392	178	122

a *A*, *B*, and *C* represent the rotational constants (in MHz); μ_*a*_, μ_*b*_, and μ_*c*_ are the absolute values of electric dipole moment components (in D); χ_*aa*_, χ_*bb*_, and χ_*cc*_ are the diagonal elements of the ^14^N nuclear quadrupole coupling tensor (in MHz);

b RMS deviation of the fit (in kHz).

c Number of measured hyperfine components.

d Relative energies (in cm^–1^) with respect to the global minimum.

e Relative energies (in cm^–1^) with respect to the global minimum, considering the zero-point energy (ZPE).

f Gibbs energies (in cm^–1^) calculated at 298 K and 1 bar.

g Standard error in parentheses in units of the last digit.

h For rotamers 1 to 4, the indicated values are from the LA-MB-FTMW experiment, while those of rotamer 5 are from the LA-CP-FTMW experiment. See Table S05 for the spectroscopic parameters obtained for the detected rotamers 1–4 using the LA-CP-FTMW technique.

Figure 1b shows that the most stable structure, *eq1*, has the aminomethyl group in the *equatorial* position. This arrangement allows for an N–H•••O=C intramolecular hydrogen bond between the amino group as the donor, and the carbonyl group as the acceptor. The second stable conformer, *ax1*, is stabilized with the same type of interaction but with the aminomethyl in the *axial* position. According to the calculations, both conformers are almost isoenergetic. Interestingly, all four next stable conformers have an *equatorial* disposition of the aminomethyl group. In this way the third most stable conformer, *eq2*, is about 100 cm^–1^ (1.2 kJ/mol) above the least energetic conformer (300 cm^–1^ (3.6 kJ/mol) if we consider the Gibbs free energy) and is stabilized by an O–H•••N hydrogen bond, with the hydroxyl group as the donor, and the nitrogen of the amine group as the acceptor. The fourth conformer, *eq3*, differs from the previous one only by a 120° twist of the CH_2_–NH group, so the acid group attacks the amino group in a different orientation, destabilizing it around 100 cm^–1^. The fifth conformer, *eq4*, is very similar to the most stable structure, but the amino group interacts with the oxygen of the acid group instead of the carbonyl one through an N–H•••O–H intramolecular hydrogen bond. It makes the molecule destabilize ∼ 200 cm^–1^ (2.4 kJ/mol) with respect to *eq1*. Finally, the sixth conformer, *eq5*, does not have any interaction between the principal functional groups of the molecule but rather is stabilized by two weak C–H•••O=C intramolecular interactions and an additional C–H•••N interaction. The next two structures, *ax2* and *ax3*, are at slightly higher energy (see the SI), and are stabilized by the same interactions as *eq4* and *eq5*, respectively, but with the ring in *axial* configuration.

### Analysis of Rotational Spectra

In the next step, we recorded the rotational spectrum of gabapentin. Gabapentin is solid at room temperature and has a high melting point (438 K), so its thermal instability prevents its vaporization using heating methods. Thus, we have produced neutral gabapentin using picosecond laser pulses in combination with a chirped-excitation Fourier transform microwave spectrometer (LA-CP-FTMW) in the 6–12 GHz range.^30–33^ The resulting broadband rotational spectrum is shown in Figure 1c and Figure S02. The spectrum has numerous rotational transitions, which anticipates that several conformers are present in the supersonic expansion. All conformers of gabapentin are near-prolate asymmetric rotors with sufficient dipole moment in the *a-axis*. Due to the characteristic patterns of μ_*a*_-type R-branch transitions, these lines were first pursued and fitted^34–38^ using rigid rotor analysis.^38^ Four conformers were located and labeled as rotamers 1–4. μ_*b*_-type transitions were also observed and measured for these four species. For rotamer 1, μ_*c*_-type transitions were also observed. Additionally, a fifth rotamer was also located, having only μ_*b*_-type transitions. All the assigned lines showed not well-resolved hyperfine structure rotational transitions due to the presence of ^14^N nuclei with electric quadrupole moment (*I* = 1). Initially, only the center of the lines was measured and fitted to a rigid rotor Hamiltonian.^38^ A total of 63, 52, 42, 60, and 11 center of lines were measured for rotamers 1 to 5. The rotational constants obtained are collected in Table S05, while Tables S06–S10 collect all the measured transitions. Aside from common fragments, no more lines remained in the spectrum, indicating that no more conformers were present.

The ^14^N nuclei of the amine group of gabapentin has quadrupole moment (*I* = 1), which interacts with the electric field gradient at the site of this nucleus, resulting in a hyperfine structure for each rotational transition.^39^ Thus, in a second step we used LA-MB-FTMW technique^19,40^ to resolve the hyperfine structure (see Figure S03 and description in the SI). The fitted values of the quadrupole coupling constants are collected in Table 1, while Tables S11–S14 collect all the measured transitions. For rotamer 5, due to its low dipole moment and abundance, it was not possible to obtain the quadrupole coupling constants.

With the information obtained we proceeded to assign the detected rotamers. The rotational and quadrupole coupling constants in Table 1 show an excellent agreement between the experimental values and those calculated using B2PLYP/6-311++G­(d,p). Note how the *axial* and *equatorial* dispositions of the ring give very different rotational constants. Thus, rotamers 1–5 correspond to the five most stable species of gabapentin, i.e., structures *eq1*, *ax1*, *eq2*, *eq3*, and *eq4*, respectively. Additionally, the observed type of transitions is in good agreement with the calculated dipole moment values. The excellent agreement between experiment and theory, highlighted by the fact that the scale factors from theory to experiment range from 0.9988 to 1.0039, supports the use of the calculated structures as accurate representations of the actual ones.

### Relative Population Abundances

The relative population abundances of the observed conformers in the supersonic jet can be estimated from relative intensity measurements,^41,42^ as the intensities of a given type of lines for a conformer are proportional to the square of the electric dipole moment component along the chosen principal inertial axis. Thus, the relative abundances were estimated by combining the relative intensities measured on different types of transitions common to all conformers with the theoretically predicted values of the electric dipole moment on the chosen axis. From these results, *eq1* and *ax1* were found to be the most stable structures, with a relative abundance of ∼75% (each structure contributing with approximately half the percentage), followed by *eq2* structure with a population of ∼20%, and *eq3* with a population of 5%. Finally, the population of *eq4* is below 1%.

### Missing Structures

Up to this point, there is a single point missing to explain the whole conformational panorama of gabapentin: the absence of the *eq5* structure. According to the calculations, its population should be like that of *eq4*, and, additionally, its dipole moment is also similar, so we should have observed this structure. To explain this anomaly, we explored the relaxed potential energy surface (PES) scan by rotating the C–C–N-H dihedral angle. It is known that when a low energy barrier separates the conformational species, the collisions with the carrier gas during the supersonic expansion provide the energy required for the conformational interconversion between different conformers.^43,44^ As shown in Figure S04, the barrier height that separates *eq5* from *eq1* is 80 cm^–1^ (0.96 kJ/mol), thus justifying the loss of this conformer during the supersonic expansion. The same occurs with *ax3* (seventh structure in stability), which relaxes to *ax1*. Finally, the absence of the eighth most stable structure, *ax2*, which is also close in energy, can be explained by being slightly less populated than *eq5*, which was already in the detection limit and has the same value of the dipole moment.

### Main Intramolecular Interactions

Once the experimental structures and the relative populations had been determined, we analyzed the key intramolecular interactions. Figure 2 shows a noncovalent interactions study (NCI)^45,46^ for the six most stable structures. The results show that *eq2* and *eq3* structures have a strong intramolecular hydrogen bond (blue color). It is not surprising because the disposition of these conformers allows a directional bond (163.9°) at a short distance (1.74 Å). These structures are also stabilized by a weak interaction C–H•••O=C, and another C–H•••O–H and C–H•••N–H from the same C–H group. In opposition to these two structures, the two most stable structures are *eq1* and *ax1*, which do not exhibit any strong intramolecular bonds. These results highlight the importance of combining quantum chemical calculations with experimental results as one would, a priori, think *eq2* and *eq3* to be the most stable structures. However, the spatial disposition of *eq1* and *ax1* allows multiple intramolecular interactions to take place: there is an N–H•••O=C hydrogen bond, as well as two C–H•••O–H interactions, a C–H•••O = C interaction and two additional C–H•••N–H intramolecular interactions. Despite all these interactions being weaker, the existence of such many interactions confers them a high stability. The least stable structure detected, *eq4*, has the same noncovalent interactions as *eq1* but with a N–H•••O–H intramolecular interaction instead of N–H•••O=C. Finally, *eq5*, which has not been experimentally detected, only exhibits weak C–H•••X interactions (X being an electronegative atom). Although not depicted in Figure 2, *ax2* structure is stabilized by the same interactions as *eq4* but with the ring in *axial* position, and *ax3* can be grouped with *eq5*. A comparison of the results using different calculations is given in the SI.

**Figure 2:**
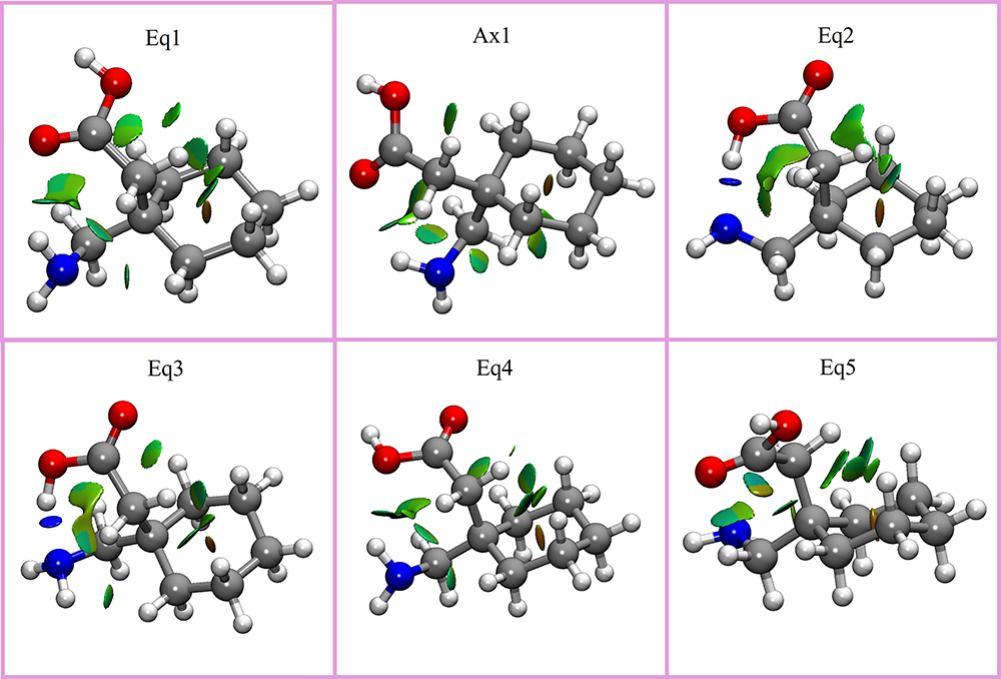
NCIPlot results of the six most stable conformers of gabapentin (only the first five have been detected). Gray corresponds to carbons, blue to nitrogen, red to oxygen, and white to hydrogen. Red surfaces correspond to repulsive forces, blue surfaces to moderate attractive forces, and green surfaces to weak attractive interactions. A contour value of 0.35 was used for the representation.

### Axial/Equatorial ratio

With all this information it is now possible to rationalize the results obtained in this work with those described in previous works, and complete the conformational preferences of gabapentin: the molecule crystallizes preferably with the aminomethyl group occupying the *axial* position.^15^ In solution, there is a rapid conformational exchange between *axial* and *equatorial* forms at room temperature, while at low temperatures, which permit conformational freezing, the most stable conformer has the aminomethyl group in the *equatorial* position, which is also the probable binding conformation of gabapentin.^15^ In isolated conditions with gabapentin being in its neutral form, we show that the two most stable conformers are similar in stability, *ax1* and *eq1*, but if we consider the population of all detected conformers, the *equatorial* disposition is predominant in an *axial*/*equatorial* = 0.37:0.63 ratio. Interestingly, this ratio is very similar to that observed in NMR experiments, where a 0.27:0.73 ratio has been determined.

### Gabapentin vs GABA

Finally, early studies indicated that due to the similarity between GABA and gabapentin, the latter could complement the former, although it was ultimately shown that they do not act on the same receptors.^4,9–13^ Thus, we decided to compare the differences in their conformational panorama. Does the incorporation of the ring affect the resulting structures and main intramolecular interactions? Fortunately, we characterized the conformational panorama of GABA using the same methodology,^47^ so the results can be compared directly. Figure 3 compares the most stable structures of gabapentin obtained in this study and those of GABA, categorized in both their stability and the main intramolecular interactions. The most stable structures of gabapentin, *eq1* and *ax1*, correspond to *gG1* and *GG3* structures of GABA, as an N–H•••O=C intramolecular hydrogen bond mainly stabilizes them. The third and fourth most stable conformers, *eq2* and *eq3*, correspond to *gG2* and *GG1* structures in GABA, as they are mainly stabilized through a O–H•••NH_2_ hydrogen bond. The fifth conformer in gabapentin, *eq4*, matches with gG3 and is stabilized with a N–H•••O–H hydrogen bond type. *ax2* structure has not been detected but also falls within this cathegory. Finally, the sixth and eight most stable conformers of gabapentin, *eq5* and *ax3*, are related to *GG2*. Most of these structures’ stabilization comes from C–H•••X interactions (X being either N or O). Despite not being detected, we decided to include *eq5*, *ax2* and *ax3*, as the GABA counterpart structures were detected, and because its absence in our experiment is due to conformational interconversion or being close in stability (in the case of *ax2*).

**Figure 3:**
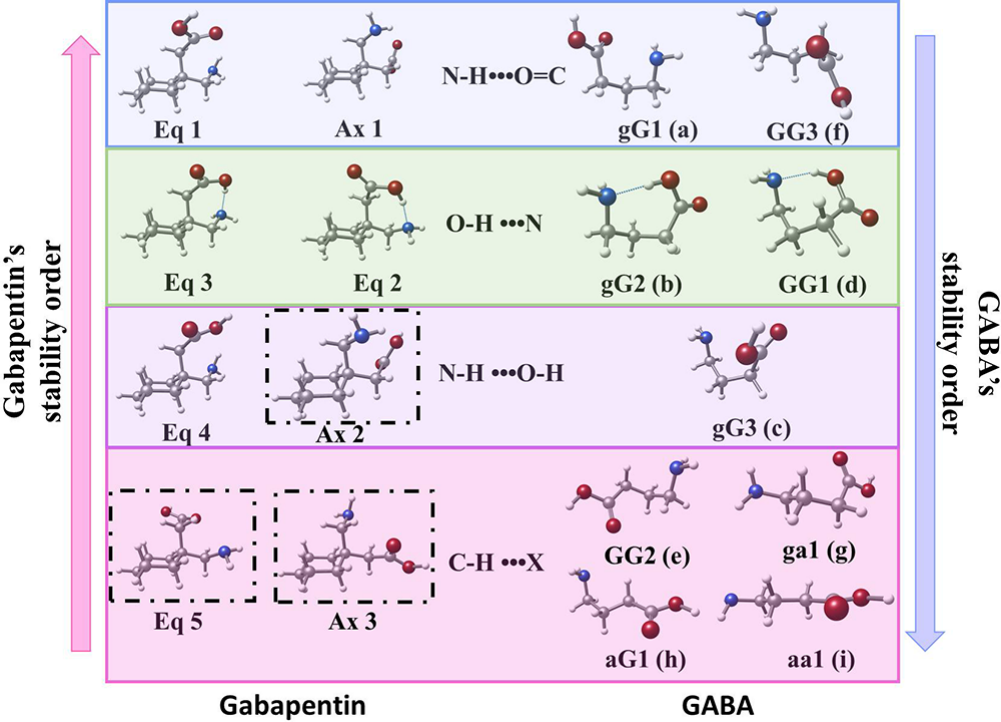
Comparison between the structures detected in gabapentin (left) and GABA^47^ (right). The arrow indicates the direction in increasing stability. The middle section shows the main stabilizing intramolecular interaction in each colored row. Black frames highlight structures not experimentally observed due to being involved in relaxation paths or being close to the sensitivity limit. Nevertheless, for comparative purposes, they have been included as they should be close in energy to *eq4*.

As can be seen in Figure 3, *eq1* and *ax1* have a relative abundance of 75%, followed by *eq2* with a population of 20%, *eq3* with a population of 5%, and *eq4* conformer with an almost negligible population (<1%). For GABA, the relative population in the supersonic jet follows the order GG2 > aG1 > gG1 > aa1 > ga1.^47^ Although no relative abundances are given for the rest of the conformers, conformers GG1, GG3, gG2 and gG3 are so weak that their population cannot be estimated, being practically negligible. The results are striking as there is a drastic change in the conformational panorama upon introducing a ring in GABA. The correlation between the relative abundances and the main intramolecular interactions is reversed entirely: for example, while almost the entire GABA’s population, around 75%, is governed by conformers stabilized only by CH•••X interactions, in gabapentin, the population with such intramolecular interactions is negligible; on the other hand, while 75% of the population in gabapentin corresponds to conformers stabilized by N–H•••C=O interactions, in GABA there is an almost negligible population of such conformers.

From a biological point of view, it could have drastic consequences: most of the GABA population is spread among structures with no strong intramolecular interactions, whereas most of the gabapentin population is. Therefore, it is not surprising that gabapentin does not interact with the same receptors as GABA. For example, it may be that gabapentin requires a larger energy contribution to break those intramolecular interactions to bind the receptor, making it energetically inefficient.

## Supplementary Material



## Notes

Differences between the B3LYP, MP2, and B2PLYP; predicted lowest-energy conformers of gabapentin; broadband rotational spectrum of gabapentin in the 6–12 GHz range; high-resolution spectrum of the 5_15_-4_14_ rotational transition using the LA-MB-FTMW spectrometer; relaxed potential energy surface from the *eq5* conformer to *eq1* conformer; spectroscopic and thermochemical parameters for the low-lying conformers of gabapentin computed at different methodologies; Cartesian coordinates in Angstroms (Å) of the conformers of gabapentin; experimental spectroscopic parameters obtained for the detected rotamers 1–5 of gabapentin using the LA-CP-FTMW technique; experimental transition frequencies (v/MHz) together with the corresponding observed-calculated differences (Δ*v*/kHz) for each species (PDF)
