# Supplementary material for: New Molecular Insights on Gabapentin
Source: ACS Phys Chem Au. 2025 Jun 3;5(4):338–45. doi: 10.1021/acsphyschemau.4c00108 (PMC12291132; doi:10.1021/acsphyschemau.4c00108)
Supplement: Supplementary file 1 [file pg4c00108_si_001.pdf]

Supporting Information

New Molecular Insights on Gabapentin

*Sofía Municio, Sergio Mato, José L. Alonso, Elena R. Alonso, Iker León**

AUTHOR INFORMATION

Corresponding Author

* **Iker León** - Grupo de Espectrocopía Molecular (GEM), Edificio Quifima, Laboratorios de Espectroscopia y Bioespectroscopia, Unidad Asociada CSIC, Parque Científico UVa, Universidad de Valladolid, 47011, Valladolid, Spain
Email: *iker.leon@uva.es*

Authors

Sofía Municio - Grupo de Espectrocopía Molecular (GEM), Edificio Quifima, Laboratorios de Espectroscopia y Bioespectroscopia, Unidad Asociada CSIC, Parque Científico UVa, Universidad de Valladolid, 47011, Valladolid, Spain
Sergio Mato - Grupo de Espectrocopía Molecular (GEM), Edificio Quifima, Laboratorios de Espectroscopia y Bioespectroscopia, Unidad Asociada CSIC, Parque Científico UVa, Universidad de Valladolid, 47011, Valladolid, Spain
José L. Alonso - Grupo de Espectrocopía Molecular (GEM), Edificio Quifima, Laboratorios de Espectroscopia y Bioespectroscopia, Unidad Asociada CSIC, Parque Científico UVa, Universidad de Valladolid, 47011, Valladolid, Spain
Elena R. Alonso - Grupo de Espectrocopía Molecular (GEM), Edificio Quifima, Laboratorios de Espectroscopia y Bioespectroscopia, Unidad Asociada CSIC, Parque Científico UVa, Universidad de Valladolid, 47011, Valladolid, Spain

Differences between the B3LYP, MP2 and B2PLYP

As we show in the main text, B2PLYP/6-311++G(d,p) predicts eigth candidate structures in the experiment, for which the five most stable structures have been detected. The absence of the sixth and seventh most stable conformers is due to conformational interconversion, and the eigthth structure is not detected probably because it fall slightly below the sensitivity limit. Therefore, we conclude that this methodology is effective.

In contrast, when using B3LYP-GD3BJ/6-311++G(d,p), the two most stable structures are *eq2* and *eq3*, followed by two other structures, *ax6* and *ax4*, that have not been detected experimentally. Moreover, the two most stable structures *eq1* and *ax1,* which have a 75% of the total population according to our results*,* become the fifth and sixth most stable structures at a considerably high relative energy. Therefore, this methodology is not consistent with the experimental results.

In the case of MP2/6-311++G(d,p), the two most stable structures detected, *eq1* and *ax1*, which have a 75% of the population, are isoenergetic to *eq2* and *eq3,* which does not agree with the experimental results either.

Figure S01. *The predicted lowest-energy conformers of gabapentin in energetical order from lowest to highest. The labelling is shown in the bottom together with the calculated $\Delta E_{ZPE}/\Delta G$ values in cm^{-1}.*

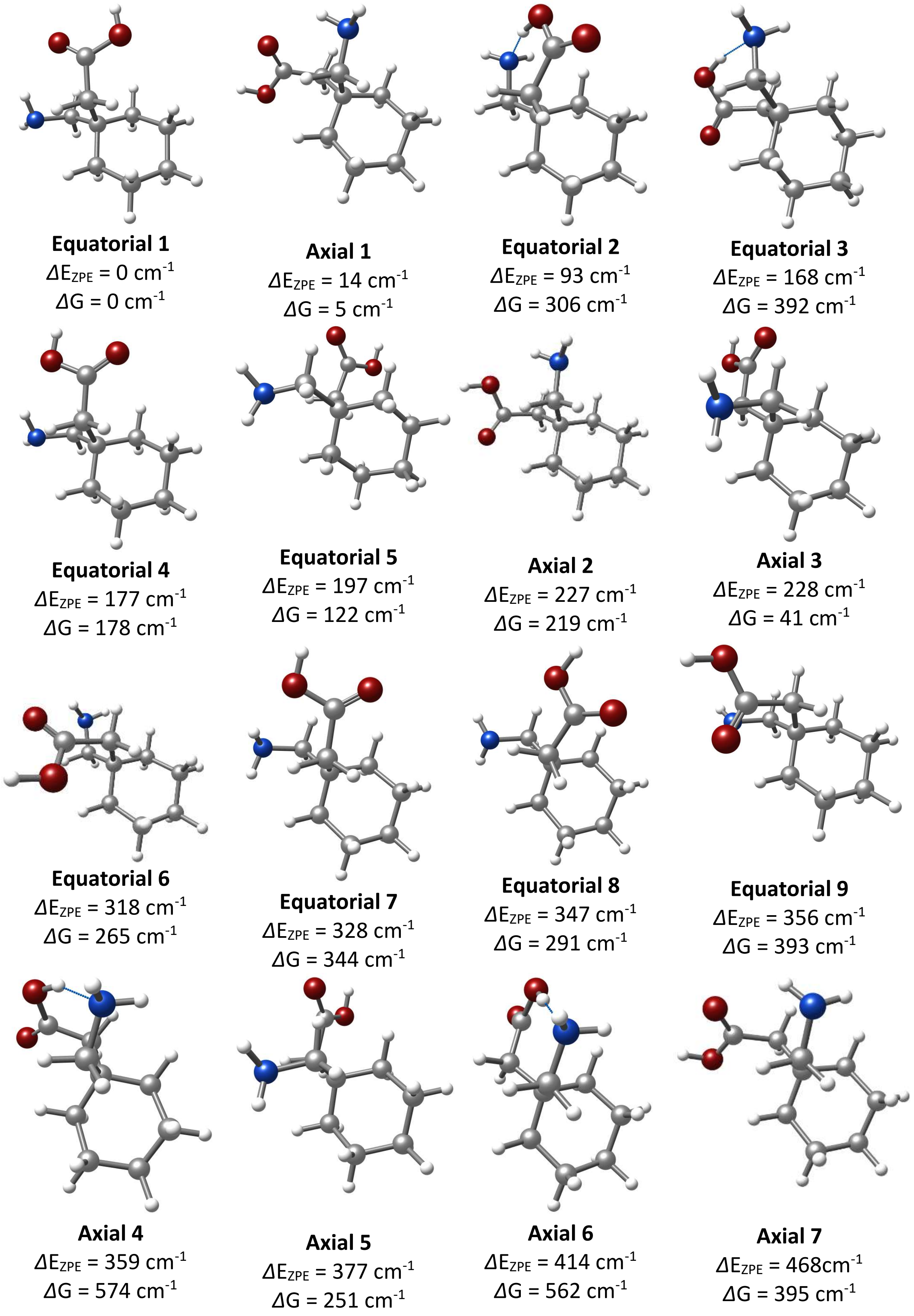

Figure S01 (Continuation). *The predicted lowest-energy conformers of gabapentin in energetical order from lowest to highest. The labelling is shown in the bottom together with the calculated $\Delta E_{ZPE}/\Delta G$ values in cm^{-1}.*

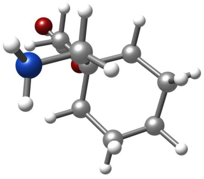

Axial 8
ΔE_{ZPE} = 537 cm^{-1}
ΔG = 380 cm^{-1}

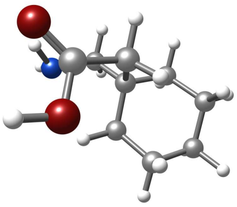

Equatorial 10
ΔE_{ZPE} = 547 cm^{-1}
ΔG = 556 cm^{-1}

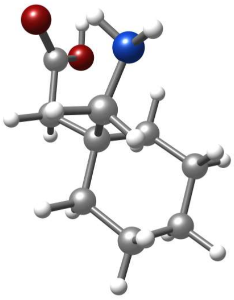

Axial 9
ΔE_{ZPE} = 573 cm^{-1}
ΔG = 497 cm^{-1}

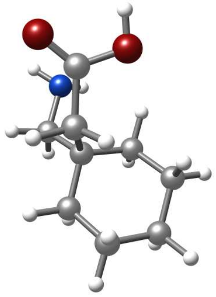

Equatorial 11
ΔE_{ZPE} = 578 cm^{-1}
ΔG = 613 cm^{-1}

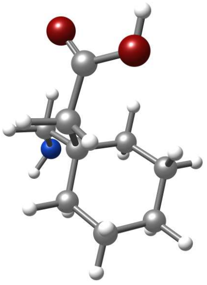

Equatorial 12
ΔE_{ZPE} = 619 cm^{-1}
ΔG = 533 cm^{-1}

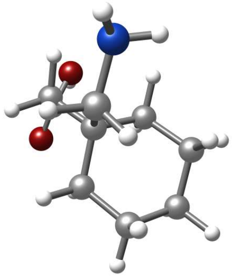

Axial 10
ΔE_{ZPE} = 646 cm^{-1}
ΔG = 579 cm^{-1}

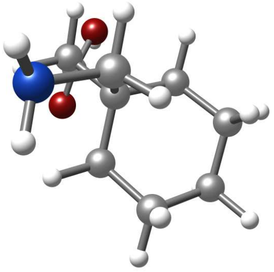

Axial 11
ΔE_{ZPE} = 648 cm^{-1}
ΔG = 516 cm^{-1}

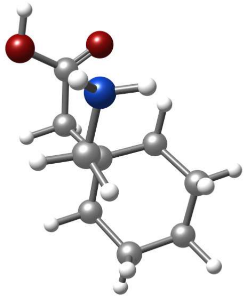

Axial 12
ΔE_{ZPE} = 655 cm^{-1}
ΔG = 664 cm^{-1}

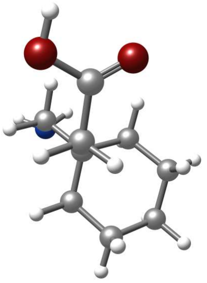

Equatorial 13
ΔE_{ZPE} = 692 cm^{-1}
ΔG = 573 cm^{-1}

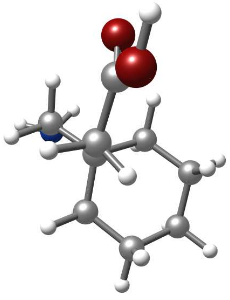

Equatorial 14
ΔE_{ZPE} = 699 cm^{-1}
ΔG = 403 cm^{-1}

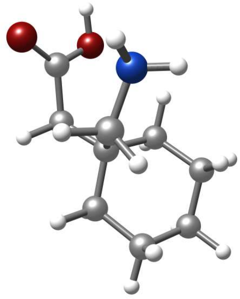

Axial 13
ΔE_{ZPE} = 706 cm^{-1}
ΔG = 729 cm^{-1}

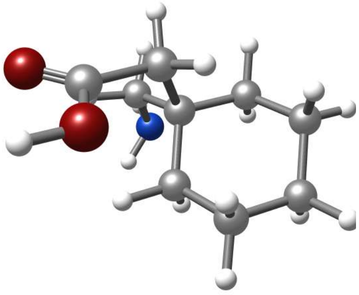

Equatorial 15
ΔE_{ZPE} = 707 cm^{-1}
ΔG = 602 cm^{-1}

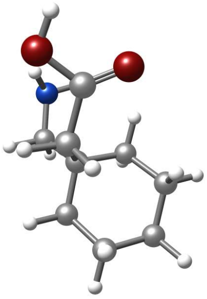

Equatorial 16
ΔE_{ZPE} = 743 cm^{-1}
ΔG = 732 cm^{-1}

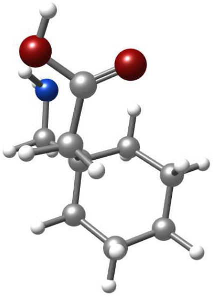

Equatorial 17
ΔE_{ZPE} = 770 cm^{-1}
ΔG = 804 cm^{-1}

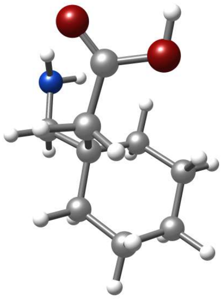

Equatorial 18
ΔE_{ZPE} = 794 cm^{-1}
ΔG = 698 cm^{-1}

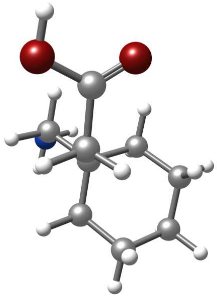

Equatorial 19
ΔE_{ZPE} = 810 cm^{-1}
ΔG = 684 cm^{-1}

Figure S01 (Continuation). *The predicted lowest-energy conformers of gabapentin in energetical order from lowest to highest. The labelling is shown in the bottom together with the calculated $\Delta E_{ZPE}/\Delta G$ values in cm^{-1}.*

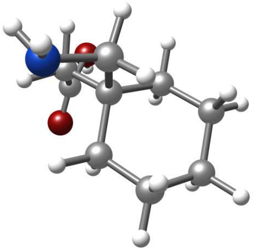

Axial 14
ΔE_{ZPE} = 814 cm^{-1}
ΔG = 720 cm^{-1}

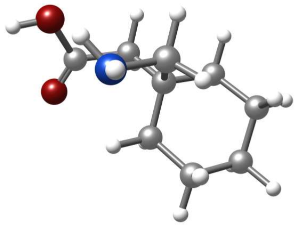

Axial 15
ΔE_{ZPE} = 892 cm^{-1}
ΔG = 866 cm^{-1}

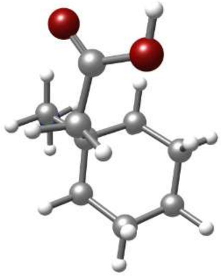

Equatorial 20
ΔE_{ZPE} = 897 cm^{-1}
ΔG = 799 cm^{-1}

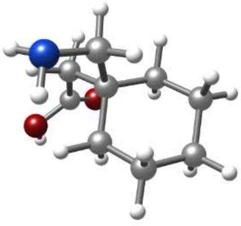

Axial 16
ΔE_{ZPE} = 899 cm^{-1}
ΔG = 804 cm^{-1}

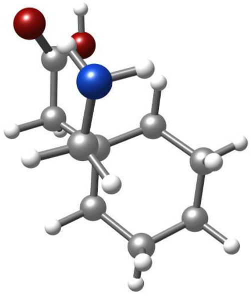

Axial 17
ΔE_{ZPE} = 931 cm^{-1}
ΔG = 802 cm^{-1}

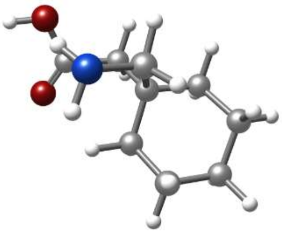

Axial 18
ΔE_{ZPE} = 991 cm^{-1}
ΔG = 921 cm^{-1}

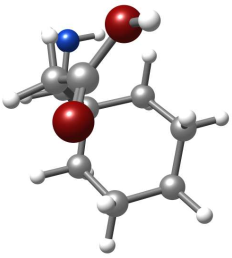

Equatorial 21
ΔE_{ZPE} = 1359 cm^{-1}
ΔG = 1362 cm^{-1}

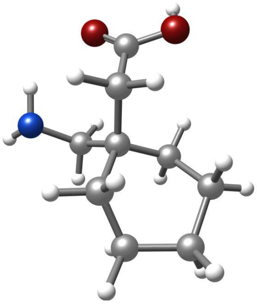

Distorted 1
ΔE_{ZPE} = 1621 cm^{-1}
ΔG = 1557 cm^{-1}

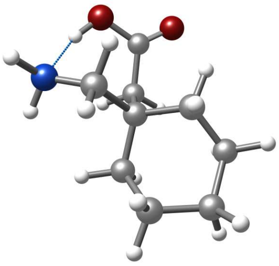

Distorted 2
ΔE_{ZPE} = 1805 cm^{-1}
ΔG = 1972 cm^{-1}

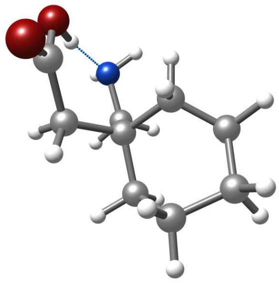

Distorted 3
ΔE_{ZPE} = 1946 cm^{-1}
ΔG = 2020 cm^{-1}

Figure S02. *Broadband rotational spectrum of gabapentin in 6-12 GHz range. The y-axis represents the intensity in arbitrary units, while the x-axis represents the frequency in MHz units. The spectrum has been expanded on the y-axis for better visualization of the lines corresponding to the rotational transitions of gabapentin*.

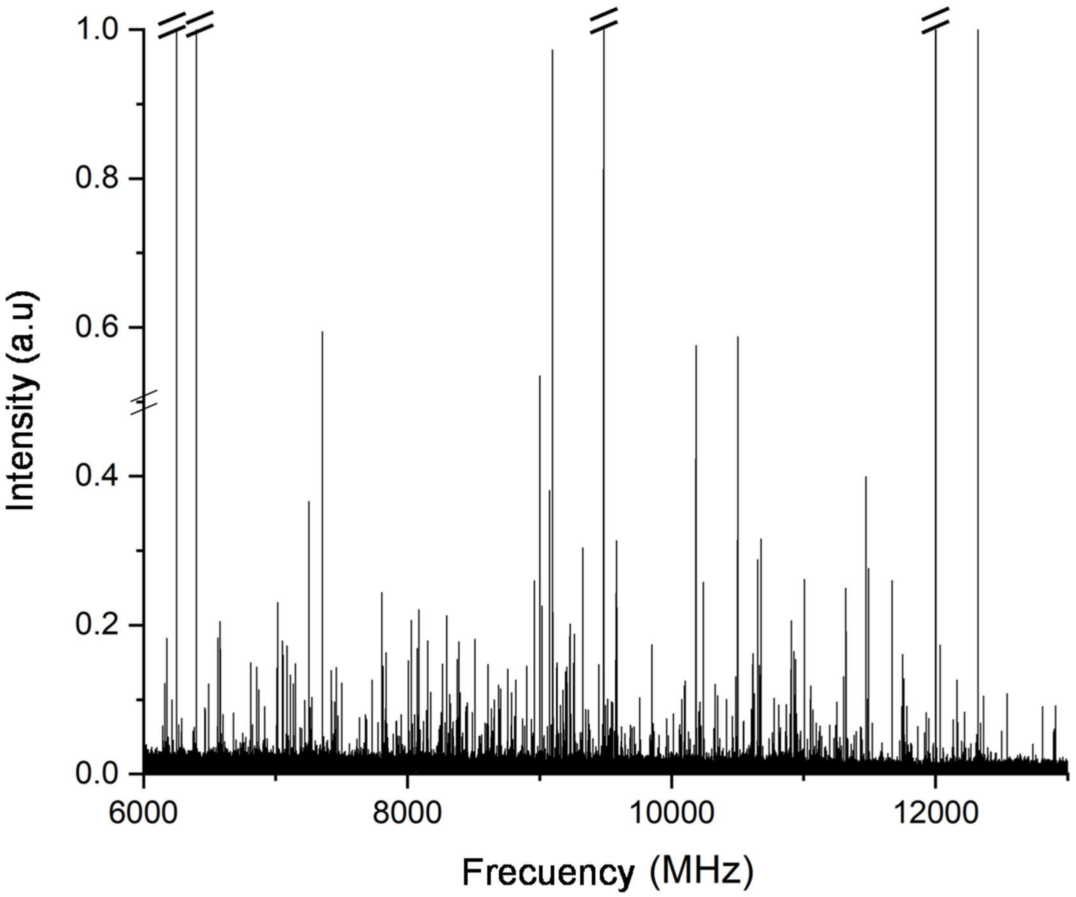

Figure S03. *An example of the comparison between the experimental and calculated $5_{15} \leftarrow 4_{14}$ rotational transition of the most stable conformer (Equatorial 1) of gabapentin showing the hyperfine components.*

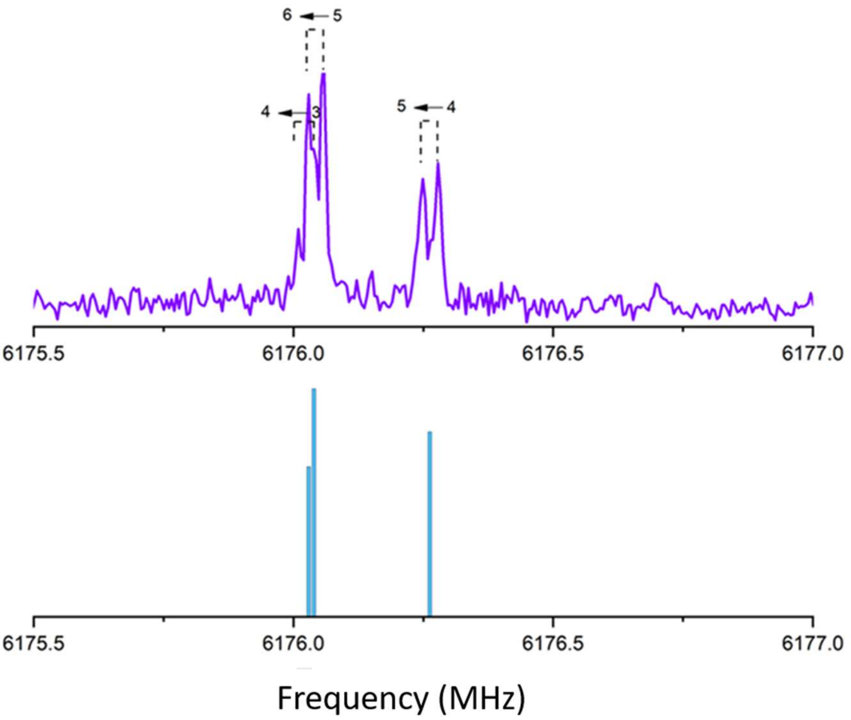

Figure S04. *Relaxed Potential Energy Surface (Relaxed-PES) by rotating the C-C-N-H dihedral angle that connects the Equatorial 5 and Equatorial 1 structures of gabapentin. The energy barrier is small enough to lose population during supersonic expansion due to the collisions with the carrier gas. The same occurs with Axial 3 structure which relaxes to Axial 1 structure by rotating the same dihedral angle.*

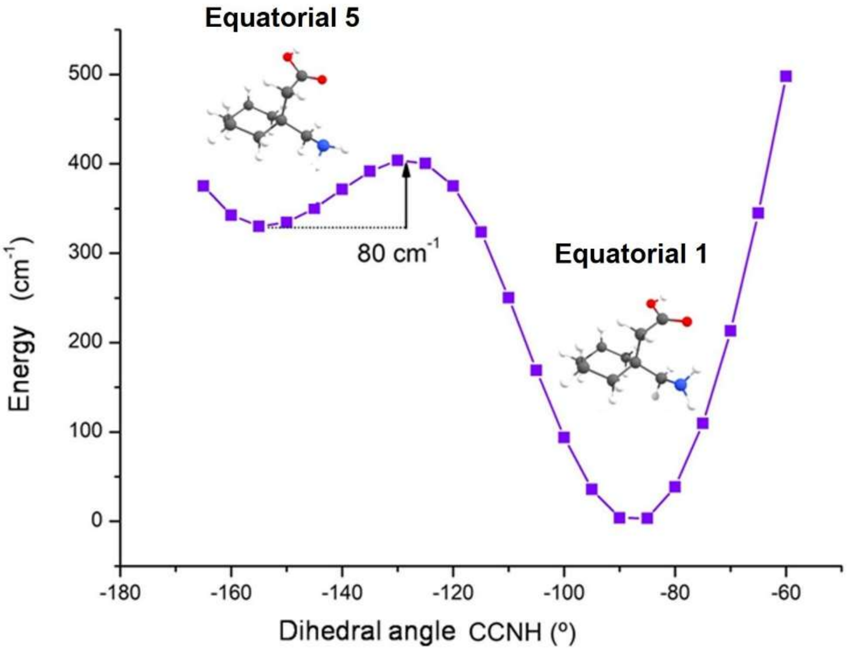

Table S01. Spectroscopic and thermochemical parameters for the low-lying conformers of gabapentin computed at *B2PLYP-GD3BJ/6-311++G(d,p)* and sorted by energy.

	Equatorial 1	Axial 1	Equatorial 2	Equatorial 3	Equatorial 4	Equatorial 5	Axial 2	Axial 3	Equatorial 6	Equatorial 7
**A(MHz)**	1277.4	1488.8	1220.2	1361.3	1316.3	1131.4	1526.8	1460.9	1275.3	1325.4
**B(MHz)**	757.4	652.1	789.8	723.3	735.8	820.1	647.9	611.5	748.3	721.5
**C(MHz)**	573.5	546.0	610.4	570.9	571.0	579.0	543.7	544.0	569.9	568.6
μ_a**(D)**	0.8	0.7	2.3	3.2	-0.6	-1.6	-0.2	-1.2	-2.2	0.9
μ_b**(D)**	-0.7	-1.0	6.3	5.5	1.4	0.3	1.4	0.4	-1.6	0.5
μ_c**(D)**	0.8	0.5	0.1	0.0	0.6	0.4	-1.0	-0.3	-0.8	-1.3
χ_{aa}	-0.0072	1.0217	1.0371	-0.0715	-0.2526	1.855	0.6634	-1.1514	1.1918	0.8022
χ_{bb}	2.2685	2.69	-2.5616	-0.6015	2.1349	1.0947	2.4994	2.7621	-0.654	0.1802
χ_{cc}	-2.2613	-3.7117	1.5244	0.673	-1.8823	-2.9497	-3.1628	-1.6107	-0.5378	-0.9824
$\Delta E(cm^{-1})$	52	70	0	23	262	327	323	421	403	418
$\Delta E_{ZPE}(cm^{-1})$	0	14	93	168	177	197	227	228	318	328
$\Delta G(cm^{-1})$	0	5	306	392	178	122	219	41	265	344

A, B, and C are the rotational constants measured in MHz. χ_{aa}, χ_{bb} *and* χ_{cc} correspond to the quadrupole coupling constants in MHz. μ_a, μ_b, *and* μ_c represent the values of the three components of the dipole moment expressed in Debyes (note that 1D ≈ $3.33564 \cdot 10^{-34}$ C · m). ΔE is the relative energy, ΔE_{ZPE} represents the relative energy including the zero-point energy correction (ZPE), and ΔG is the Gibbs free energy at 1 atm and a temperature of 298 K.

Table S01 (Continuation). Spectroscopic and thermochemical parameters for the low-lying conformers of gabapentin computed at *B2PLYP-GD3BJ/6-311++G(d,p)* and sorted by energy.

	Equatorial 8	Equatorial 9	Axial 4	Axial 5	Axial 6	Axial 7	Axial 8	Equatorial 10	Axial 9	Equatorial 11
A(MHz)	1140.0	1425.2	1553.4	1384.1	1465.8	1484.5	1226.2	1375.1	1597.4	1444.0
B(MHz)	818.6	671.5	641.4	663.1	634.1	649.6	695.4	703.1	605.9	665.4
C(MHz)	579.4	608.3	547.1	549.7	577.9	543.8	554.9	605.7	582.2	611.9
μ_a(D)	0.5	-0.7	-5.1	1.8	2.1	-1.3	-1.1	-1.3	1.2	1.9
μ_b(D)	2.4	2.1	4.3	-0.4	1.7	-2.0	1.4	0.4	-0.2	1.0
μ_c(D)	1.2	-0.3	0.3	-0.1	0.6	1.5	0.6	0.0	0.3	1.4
χ_{aa}	1.6875	1.1104	-3.105	0.0128	-2.7189	2.131	-4.5566	2.2795	2.0221	1.8696
χ_{bb}	1.0742	-2.1593	1.7049	2.7624	0.6137	0.3291	2.6234	-1.5258	-1.0277	-4.1701
χ_{cc}	-2.7617	1.0489	1.4002	-2.7752	2.1052	-2.4602	1.9332	-0.7537	-0.9944	2.3005
$\Delta E(cm^{-1})$	470	506	235	516	346	571	728	639	663	741
$\Delta E_{ZPE}(cm^{-1})$	347	356	359	377	414	468	537	547	573	578
$\Delta G(cm^{-1})$	291	393	574	251	562	395	380	556	497	613

A, B, and C are the rotational constants measured in MHz. χ_{aa}, χ_{bb} *and* χ_{cc} correspond to the quadrupole coupling constants in MHz. μ_a, μ_b, *and* μ_c represent the values of the three components of the dipole moment expressed in Debyes (note that 1D ≈ $3.33564 \cdot 10^{-34}$ C · m). ΔE is the relative energy, ΔE_{ZPE} represents the relative energy including the zero-point energy correction (ZPE), and ΔG is the Gibbs free energy at 1 atm and a temperature of 298 K.

Table S01 (Continuation). Spectroscopic and thermochemical parameters for the low-lying conformers of gabapentin computed at *B2PLYP-GD3BJ/6-311++G(d,p)* and sorted by energy.

	Equatorial 12	Axial 10	Axial 11	Axial 12	Equatorial 13	Equatorial 14	Axial 13	Equatorial 15	Equatorial 16	Equatorial 17
A(MHz)	1127.0	1153.4	1152.6	1706.1	1202.7	1117.3	1704.0	1122.0	1376.3	1378.6
B(MHz)	792.3	730.5	718.6	586.5	712.4	745.9	591.5	793.0	687.8	700.8
C(MHz)	582.5	598.9	594.0	579.4	582.7	577.0	582.5	585.8	601.2	602.4
$\mu_a(D)$	0.9	-2.0	1.9	1.6	0.6	-0.4	2.3	0.4	0.7	-0.1
$\mu_b(D)$	-0.1	1.4	0.5	-1.4	1.3	0.0	0.8	-0.3	-0.3	2.1
$\mu_c(D)$	-0.7	0.6	-1.6	1.5	0.5	-0.6	0.5	-0.3	-1.2	-1.2
χ_{aa}	0.9116	-4.8983	-4.3295	-1.9097	-3.801	-3.178	-0.4925	-3.9065	2.1355	2.5344
χ_{bb}	0.312	2.684	2.5666	2.3417	2.5696	1.2474	2.4933	1.7709	-4.2737	-2.0643
χ_{cc}	-1.2236	2.2142	1.7628	-0.432	1.2314	1.9305	-2.0009	2.1356	2.1382	-0.47
$\Delta E(cm^{-1})$	759	764	793	805	845	903	875	843	867	906
$\Delta E_{ZPE}(cm^{-1})$	619	646	648	655	692	699	706	707	743	770
$\Delta G(cm^{-1})$	533	579	516	664	573	403	729	602	732	804

A, B, and C are the rotational constants measured in MHz. χ_{aa}, χ_{bb} *and* χ_{cc} correspond to the quadrupole coupling constants in MHz. μ_a, μ_b, *and* μ_c represent the values of the three components of the dipole moment expressed in Debyes (note that 1D ≈ $3.33564 \cdot 10^{-34}$ C · m). ΔE is the relative energy, ΔE_{ZPE} represents the relative energy including the zero-point energy correction (ZPE), and ΔG is the Gibbs free energy at 1 atm and a temperature of 298 K.

Table S01 (Continuation). Spectroscopic and thermochemical parameters for the low-lying conformers of gabapentin computed at *B2PLYP-GD3BJ/6-311++G(d,p)* and sorted by energy.

	Equatorial 18	Equatorial 19	Axial 14	Axial 15	Equatorial 20	Axial 16	Axial 17	Axial 18	Equatorial 21	Distorted 1
A(MHz)	1296.9	1191.4	1142.7	1619.1	1104.9	1146.7	1510.3	1630.2	1009.1	1419.5
B(MHz)	731.6	715.9	724.7	599.0	801.2	727.4	621.5	591.1	891.3	694.4
C(MHz)	599.8	580.3	592.8	576.9	583.9	595.2	577.9	578.0	655.2	531.8
μ_a***(D)***	2.2	-1.3	-0.7	-0.6	1.9	0.0	1.3	0.6	-2.3	-0.7
μ_b***(D)***	-2.0	-0.1	1.1	2.2	-2.3	0.1	-2.6	-0.2	0.9	-0.9
μ_c***(D)***	0.2	1.1	2.3	1.2	0.1	1.3	-0.1	-1.2	1.1	-0.8
χ_{aa}	1.9447	-0.5734	2.1549	2.3058	1.2293	-0.2392	2.0828	2.361	0.24	0.5306
χ_{bb}	-4.506	-1.6887	2.1665	-2.1279	-3.5676	-0.8345	-4.833	-4.4918	-2.6181	2.576
χ_{cc}	2.5614	2.2621	-4.3214	-0.1778	2.3383	1.0737	2.7502	2.1308	2.3782	-3.1066
$\Delta E(cm^{-1})$	866	969	964	1021	1051	1051	1011	1124	1464	1697
$\Delta E_{ZPE}(cm^{-1})$	794	810	814	892	897	899	931	991	1359	1621
$\Delta G(cm^{-1})$	698	684	720	866	799	804	802	921	1362	1557

A, B, and C are the rotational constants measured in MHz. χ_{aa}, χ_{bb} *and* χ_{cc} correspond to the quadrupole coupling constants in MHz. μ_a, μ_b, *and* μ_c represent the values of the three components of the dipole moment expressed in Debyes (note that 1D ≈ $3.33564 \cdot 10^{-34}$ C · m). ΔE is the relative energy, ΔE_{ZPE} represents the relative energy including the zero-point energy correction (ZPE), and ΔG is the Gibbs free energy at 1 atm and a temperature of 298 K.

Table S01 (Continuation). Spectroscopic and thermochemical parameters for the low-lying conformers of gabapentin computed at *B2PLYP-GD3BJ/6-311++G(d,p)* and sorted by energy.

	Distorted 2	Distorted 3
A(MHz)	1483.5	1299.2
B(MHz)	680.4	699.5
C(MHz)	532.7	586.0
μ_a***(D)***	4.3	-3.9
μ_b***(D)***	4.9	5.6
μ_c***(D)***	0.4	1.3
χ_{aa}	-1.7956	-0.5162
χ_{bb}	0.4706	-1.5095
χ_{cc}	1.325	2.0257
$\Delta E(cm^{-1})$	1706	1915
$\Delta E_{ZPE}(cm^{-1})$	1805	1946
$\Delta G(cm^{-1})$	1972	2020

A, B, and C are the rotational constants measured in MHz. χ_{aa}, χ_{bb} and χ_{cc} correspond to the quadrupole coupling constants in MHz. μ_a, μ_b, *and* μ_c represent the values of the three components of the dipole moment expressed in Debyes (note that 1D ≈ $3.33564 \cdot 10^{-34}$ C · m). ΔE is the relative energy, ΔE_{ZPE} represents the relative energy including the zero-point energy correction (ZPE), and ΔG is the Gibbs free energy at 1 atm and a temperature of 298 K.

Table S02. Spectroscopic and thermochemical parameters for the low-lying conformers of gabapentin computed at *B3LYP-GD3BJ/6-311++G(d,p)* and sorted by energy.

	Equatorial 2	Equatorial 3	Axial 6	Axial 4	Equatorial 1	Axial 1	Equatorial 4	Equatorial 5	Axial 2	Ecautorial 6
A(MHz)	1219.0	1358.6	1471.2	1553.1	1278.8	1490.3	1315.4	1123.1	1526.4	1278.5
B(MHz)	785.2	720.1	629.2	638.0	752.2	648.6	731.8	816.5	644.9	742.7
C(MHz)	608.3	569.1	574.5	545.0	571.4	543.7	569.3	578.2	541.8	568.1
μ_a***(D)***	-2.3	-3.3	-5.7	-5.2	-0.9	0.8	0.5	1.6	-0.2	-2.3
μ_b***(D)***	6.4	5.6	4.3	4.3	0.7	1.0	1.4	0.4	1.5	-1.6
μ_c***(D)***	-0.1	0.0	-1.4	0.3	0.8	-0.5	-0.5	-0.2	-1.0	-0.8
χ_{aa}	1.01	-0.1053	-2.778	-3.2041	-0.0153	1.0606	-0.2703	1.8962	0.6963	1.2201
χ_{bb}	-2.6199	-0.6899	0.6289	1.6978	2.3354	2.7546	2.1848	0.9855	2.5544	-0.6285
χ_{cc}	1.61	0.7951	2.1491	1.5063	-2.3201	-3.8152	-1.9145	-2.8817	-3.2506	-0.5917
$\Delta E(cm^{-1})$	0	123	376	334	607	636	808	866	882	882
$\Delta E_{ZPE}(cm^{-1})$	0	168	345	359	453	481	626	636	684	701
$\Delta G(cm^{-1})$	0	180	279	366	224	251	411	310	452	424

A, B, and C are the rotational constants measured in MHz. χ_{aa}, χ_{bb} *and* χ_{cc} correspond to the quadrupole coupling constants in MHz. μ_a, μ_b, *and* μ_c represent the values of the three components of the dipole moment expressed in Debyes (note that 1D ≈ $3.33564 \cdot 10^{-34}$ C · m). ΔE is the relative energy, ΔE_{ZPE} represents the relative energy including the zero-point energy correction (ZPE), and ΔG is the Gibbs free energy at 1 atm and a temperature of 298 K.

Table S02 (Continuation). Spectroscopic and thermochemical parameters for the low-lying conformers of gabapentin computed at *B3LYP-GD3BJ/6-311++G(d,p)* and sorted by energy. Axial 2* converges into Axial 2 with B2PLYP and MP2.

	Equatorial 7	Axial 3	Equatorial 8	Axial 5	Equatorial 9	Axial 7	Axial 2*	Equatorial 10	Axial 9	Equatorial 16
A(MHz)	1323.6	1456.1	1137.0	1384.7	1421.1	1485.7	1537.5	1371.7	1594.9	1379.4
B(MHz)	718.5	610.7	816.1	656.4	669.6	646.5	639.5	701.5	603.9	684.0
C(MHz)	567.2	542.8	578.3	547.7	606.4	541.8	540.8	604.0	580.0	600.1
μ_a***(D)***	-0.9	-1.2	-0.5	-1.7	0.7	1.4	-0.9	1.4	1.3	-0.7
μ_b***(D)***	0.6	0.4	2.5	-0.3	2.1	2.0	0.7	0.4	-0.2	-0.2
μ_c***(D)***	1.3	-0.4	-1.2	0.0	0.3	1.5	-1.3	-0.1	0.3	1.2
χ_{aa}	0.842	-1.1502	1.7574	-0.0315	1.0909	2.2006	2.2255	2.3239	2.0909	2.2142
χ_{bb}	0.1709	2.8082	1.0205	2.7895	-1.8835	0.3437	1.154	-1.5275	-1.0635	-4.3651
χ_{cc}	-1.0129	-1.658	-2.7779	-2.7581	0.7927	-2.5443	-3.3795	-0.7964	-1.0273	2.1509
$\Delta E(cm^{-1})$	892	1093	1026	1087	1101	1067	1103	1185	1213	1265
$\Delta E_{ZPE}(cm^{-1})$	712	804	806	845	856	869	901	1001	1031	1058
$\Delta G(cm^{-1})$	512	408	535	468	695	578	680	802	752	856

A, B, and C are the rotational constants measured in MHz. χ_{aa}, χ_{bb} *and* χ_{cc} correspond to the quadrupole coupling constants in MHz. μ_a, μ_b,*and* μ_c represent the values of the three components of the dipole moment expressed in Debyes (note that 1D ≈ $3.33564 \cdot 10^{-34}$ C · m). ΔE is the relative energy, ΔE_{ZPE} represents the relative energy including the zero-point energy correction (ZPE), and ΔG is the Gibbs free energy at 1 atm and a temperature of 298 K.

Table S02 (Continuation). Spectroscopic and thermochemical parameters for the low-lying conformers of gabapentin computed at *B3LYP-GD3BJ/6-311++G(d,p)* and sorted by energy.

	Equatorial 11	Equatorial 12	Axial 8	Axial 10	Equatorial 18	Axial 11	Axial 12	Equatorial 13	Equatorial 15	Equatorial 19
A(MHz)	1438.2	1120.5	1223.6	1150.6	1305.0	1151.9	1709.6	1200.4	1117.3	1189.1
B(MHz)	664.4	792.0	693.4	727.7	724.7	714.7	582.9	708.1	791.6	711.5
C(MHz)	609.8	580.8	553.4	595.9	598.7	590.8	574.6	580.6	583.5	578.6
$\mu_a(D)$	1.9	-1.0	-1.1	-2.0	2.3	-1.9	-1.6	0.5	-0.4	-1.3
$\mu_b(D)$	1.0	-0.1	1.4	1.5	-1.9	0.6	-1.5	1.3	-0.4	-0.1
$\mu_c(D)$	1.5	0.7	0.6	0.6	0.2	1.6	-1.4	0.5	0.3	1.2
χ_{aa}	1.9362	1.0963	-4.7642	-5.0744	2.0153	-4.5147	-2.1012	-3.8092	-3.963	-0.5271
χ_{bb}	-4.2224	0.2984	2.6943	2.7549	-4.6345	2.6336	2.4169	2.6487	1.8041	-1.8155
χ_{cc}	2.2862	-1.3947	2.0699	2.3195	2.6192	1.8811	-0.3156	1.1605	2.1589	2.3426
$\Delta E(cm^{-1})$	1345	1335	1394	1349	1314	1379	1407	1425	1429	1446
$\Delta E_{ZPE}(cm^{-1})$	1069	1094	1116	1133	1137	1140	1154	1175	1188	1195
$\Delta G(cm^{-1})$	877	777	767	835	735	783	945	841	838	854

A, B, and C are the rotational constants measured in MHz. χ_{aa}, χ_{bb} *and* χ_{cc} correspond to the quadrupole coupling constants in MHz. μ_a, μ_b, *and* μ_c represent the values of the three components of the dipole moment expressed in Debyes (note that 1D ≈ $3.33564 \cdot 10^{-34}$ C · m). ΔE is the relative energy, ΔE_{ZPE} represents the relative energy including the zero-point energy correction (ZPE), and ΔG is the Gibbs free energy at 1 atm and a temperature of 298 K.

Table S02 (Continuation). Spectroscopic and thermochemical parameters for the low-lying conformers of gabapentin computed at *B3LYP-GD3BJ/6-311++G(d,p)* and sorted by energy.

	**Equatorial 17**	**Axial 13**	**Equatorial 20**	**Equatorial 14**	**Axial 16**	**Axial 17**	**Axial 14**	**Axial 18**	**Axial 15**	**Equatorial 21**
A(MHz)	1375.3	1701.5	1099.5	1121.4	1143.8	1504.7	1141.5	1636.9	1617.0	1008.0
B(MHz)	699.3	589.1	801.0	735.7	724.8	620.3	721.1	587.8	597.1	888.4
C(MHz)	600.9	580.3	582.0	574.3	592.3	575.4	589.9	575.4	574.9	653.2
μ_a***(D)***	0.1	2.4	-1.9	0.4	-0.1	-1.3	-0.7	-0.6	-0.6	-2.3
μ_b***(D)***	2.2	-0.9	-2.3	0.0	0.2	-2.7	1.2	-0.1	2.2	-0.9
μ_c***(D)***	1.2	-0.5	0.0	0.7	-1.3	0.1	2.3	1.2	1.2	-1.1
χ_{aa}	2.5814	-0.4285	1.3527	-3.2932	-0.2926	2.125	2.2501	2.4427	2.3769	0.263
χ_{bb}	-2.0553	2.4977	-3.7537	1.399	-0.8313	-4.9295	2.1697	-4.2654	-2.1365	-2.7052
χ_{cc}	-0.5261	-2.0692	2.401	1.8942	1.1239	2.8045	-4.4198	1.8227	-0.2405	2.4422
$\Delta E(cm^{-1})$	1421	1488	1505	1569	1529	1472	1556	1549	1547	1920
$\Delta E_{ZPE}(cm^{-1})$	1196	1204	1257	1265	1285	1296	1304	1320	1332	1717
$\Delta G(cm^{-1})$	1022	984	935	760	964	935	977	1051	1111	1497

A, B, and C are the rotational constants measured in MHz. χ_{aa}, χ_{bb} and χ_{cc} correspond to the quadrupole coupling constants in MHz. μ_a, μ_b,*and* μ_c represent the values of the three components of the dipole moment expressed in Debyes (note that 1D ≈ $3.33564 \cdot 10^{-34}$ C · m). ΔE is the relative energy, ΔE_{ZPE} represents the relative energy including the zero-point energy correction (ZPE), and ΔG is the Gibbs free energy at 1 atm and a temperature of 298 K.

Table S02 (Continuation). Spectroscopic and thermochemical parameters for the low-lying conformers of gabapentin computed at *B3LYP-GD3BJ/6-311++G(d,p)* and sorted by energy.

	Distorted 2	Equatorial 22	Distorted 3	Distorted 1
A(MHz)	1481.2	1017.1	1301.3	1420.2
B(MHz)	677.1	855.2	693.8	690.5
C(MHz)	530.8	650.8	582.9	530.0
$\mu_a(D)$	-4.4	-1.7	-4.0	0.8
$\mu_b(D)$	5.0	-1.0	5.7	-0.9
$\mu_c(D)$	-0.4	-1.5	1.3	0.8
χ_{aa}	-1.8693	-0.4668	-0.6173	0.5267
χ_{bb}	0.4462	-1.7358	-1.4807	2.6412
χ_{cc}	1.4231	2.2027	2.098	-3.1679
$\Delta E(cm^{-1})$	1745	2017	1928	2201
$\Delta E_{ZPE}(cm^{-1})$	1738	1791	1859	2021
$\Delta G(cm^{-1})$	1679	1382	1710	1732

A, B, and C are the rotational constants measured in MHz. χ_{aa}, χ_{bb} and χ_{cc} correspond to the quadrupole coupling constants in MHz. μ_a, μ_b, *and* μ_c represent the values of the three components of the dipole moment expressed in Debyes (note that 1D ≈ $3.33564 \cdot 10^{-34}$ C · m). ΔE is the relative energy, ΔE_{ZPE} represents the relative energy including the zero-point energy correction (ZPE), and ΔG is the Gibbs free energy at 1 atm and a temperature of 298 K.

Table S03. Spectroscopic and thermochemical parameters for the low-lying conformers of gabapentin computed at *MP2-GD3BJ/6-311++G(d,p)* and sorted by energy.

	Equatorial 3	Equatorial 2	Equatorial 1	Axial 1	Equatorial 4	Axial 5	Equatorial 5	Axial 2	Equatorial 8	Equatorial 7
**A(MHz)**	1368.0	1224.9	1276.3	1485.6	1318.7	1555.9	1145.3	1529.9	1142.5	1329.1
**B(MHz)**	731.1	798.7	768.4	659.8	744.5	648.8	825.1	654.1	827.1	728.9
**C(MHz)**	575.8	615.1	578.6	551.3	575.4	552.1	581.6	548.1	582.9	572.7
**μ_a(D)**	3.1	2.1	0.7	-0.6	-0.6	-5.0	-1.5	-0.1	0.4	0.9
**μ_b(D)**	5.3	6.2	-0.6	-0.9	1.2	4.1	0.3	1.3	2.3	0.4
**μ_c(d)**	0.0	0.1	0.8	-0.6	0.6	0.3	0.5	-1.0	1.2	-1.3
**χ_{aa}**	-0.0299	1.0839	-0.0314	0.9272	-0.2683	-2.9294	1.7246	0.5533	1.5289	0.6959
**χ_{bb}**	-0.492	-2.4367	2.1166	2.5665	2.0035	1.6947	1.1778	2.3866	1.1203	0.1939
**χ_{cc}**	0.5219	1.3529	-2.0851	-3.4937	-1.7352	1.2348	-2.9023	-2.94	-2.6491	-0.8898
**ΔE(cm^{-1})**	0	75	209	323	407	285	543	533	680	682
**ΔE_{ZPE}(cm^{-1})**	0	16	23	127	177	264	267	295	410	432
**ΔG(cm^{-1})**	164	181	0	76	131	414	171	243	307	394

A, B, and C are the rotational constants measured in MHz. χ_{aa}, χ_{bb} *and* χ_{cc} correspond to the quadrupole coupling constants in MHz. μ_a, μ_b, *and* μ_c represent the values of the three components of the dipole moment expressed in Debyes (note that 1D ≈ $3.33564 \cdot 10^{-34}$ C · m). ΔE is the relative energy, ΔE_{ZPE} represents the relative energy including the zero-point energy correction (ZPE), and ΔG is the Gibbs free energy at 1 atm and a temperature of 298 K.

Table S03 (Continuation). Spectroscopic and thermochemical parameters for the low-lying conformers of gabapentin computed at *MP2-GD3BJ/6-311++G(d,p)* and sorted by energy.

	Equatorial 6	Axial 7	Equatorial 9	Axial 6	Equatorial 11	Axial 3	Equatorial 10	Axial 8	Equatorial 12	Axial 10
**A(MHz)**	1271.0	1462.5	1440.5	1462.2	1459.2	1540.8	1386.0	1480.8	1137.0	1604.4
**B(MHz)**	760.3	643.2	674.4	615.6	667.8	648.1	705.3	656.7	795.8	609.8
**C(MHz)**	574.7	585.1	613.5	546.9	617.1	546.9	610.1	548.6	586.5	587.8
**μ_a(D)**	-2.1	-5.3	-0.6	1.2	1.7	0.8	-1.1	-1.2	0.8	1.1
**μ_b(D)**	-1.7	4.3	2.2	0.4	1.0	0.5	0.5	-1.9	0.0	-0.1
**μ_c(d)**	-0.9	-1.2	-0.2	0.2	1.3	1.3	0.0	1.5	-0.6	0.3
**χ_{aa}**	1.0981	-2.416	1.1814	-1.1618	1.7724	1.9837	2.1934	1.9832	0.658	1.8641
**χ_{bb}**	-0.6714	0.4879	-2.6866	2.6752	-4.0304	1.1266	-1.632	0.2765	0.3555	-0.9245
**χ_{cc}**	-0.4267	1.9281	1.5052	-1.5134	2.2581	-3.1103	-0.5614	-2.2597	-1.0136	-0.9396
**ΔE(cm^{-1})**	667	551	845	981	932	947	939	941	1021	1032
**ΔE_{ZPE}(cm^{-1})**	438	476	545	636	651	678	684	687	740	777
**ΔG(cm^{-1})**	353	572	505	352	660	629	620	563	617	611

A, B, and C are the rotational constants measured in MHz. χ_{aa}, χ_{bb} *and* χ_{cc} correspond to the quadrupole coupling constants in MHz. μ_a, μ_b, *and* μ_c represent the values of the three components of the dipole moment expressed in Debyes (note that 1D ≈ $3.33564 \cdot 10^{-34}$ C · m). ΔE is the relative energy, ΔE_{ZPE} represents the relative energy including the zero-point energy correction (ZPE), and ΔG is the Gibbs free energy at 1 atm and a temperature of 298 K.

Table S03 (Continuation). Spectroscopic and thermochemical parameters for the low-lying conformers of gabapentin computed at *MP2-GD3BJ/6-311++G(d,p)* and sorted by energy.

	Axial 11	Axial 14	Axial 13	Equatorial 14	Axial 12	Equatorial 13	Equatorial 17	Equatorial 16	Axial 15	Axial 9
**A(MHz)**	1161.8	1713.6	1709.1	1128.4	1158.8	1206.3	1386.3	1376.8	1147.3	1236.3
**B(MHz)**	736.1	597.2	594.3	798.4	725.0	721.3	704.5	693.8	731.7	697.9
**C(MHz)**	605.7	586.8	587.3	590.9	599.9	587.9	606.6	604.9	598.6	558.0
**μ_a(D)**	-2.0	2.2	1.7	0.2	1.9	0.7	-0.2	0.7	-0.7	-1.1
**μ_b(D)**	1.3	0.8	-1.0	-0.3	0.4	1.3	2.0	-0.5	1.0	1.3
**μ_c(d)**	0.5	0.5	1.9	0.3	-1.6	0.4	-1.2	-1.1	2.3	0.6
**χ_{aa}**	-4.5868	-0.5911	-1.5981	-3.7389	-3.9932	-3.6698	2.4295	1.9791	1.9626	-4.2265
**χ_{bb}**	2.5445	2.4729	2.4093	1.6854	2.4195	2.4294	-2.056	-4.0245	2.1505	2.4836
**χ_{cc}**	2.0424	-1.8818	-0.8113	2.0535	1.5737	1.2405	-0.3734	2.0454	-4.1131	1.7429
**ΔE(cm^{-1})**	1043	1082	1083	1093	1127	1152	1159	1241	1243	1311
**ΔE_{ZPE}(cm^{-1})**	787	797	800	820	832	848	858	948	953	955
**ΔG(cm^{-1})**	702	818	799	702	657	675	827	852	838	718

A, B, and C are the rotational constants measured in MHz. χ_{aa}, χ_{bb} and χ_{cc} correspond to the quadrupole coupling constants in MHz. μ_a, μ_b,*and* μ_c represent the values of the three components of the dipole moment expressed in Debyes (note that 1D ≈ $3.33564 \cdot 10^{-34}$ C · m). ΔE is the relative energy, ΔE_{ZPE} represents the relative energy including the zero-point energy correction (ZPE), and ΔG is the Gibbs free energy at 1 atm and a temperature of 298 K.

Table S03 (Continuation). Spectroscopic and thermochemical parameters for the low-lying conformers of gabapentin computed at *MP2-GD3BJ/6-311++G(d,p)* and sorted by energy.

	Equatorial 18	Equatorial 19	Axial 16	Equatorial 20	Axial 17	Axial 18	Axial 20	Equatorial 21	Equatorial 22
**A(MHz)**	1303.6	1196.6	1625.6	1112.3	1155.7	1523.2	1632.2	1013.4	1036.2
**B(MHz)**	734.0	724.2	602.8	805.1	732.9	624.0	595.5	898.8	859.4
**C(MHz)**	603.4	584.7	582.0	588.4	601.8	583.6	585.4	660.1	653.2
**μ_a(D)**	2.1	-1.3	-0.5	1.9	0.0	1.2	0.6	-2.1	-1.7
**μ_b(D)**	-2.0	-0.2	2.1	-2.3	0.0	-2.6	-0.4	1.0	0.8
**μ_c(d)**	0.2	1.1	1.1	0.1	1.3	-0.2	-1.1	1.0	1.6
**χ_{aa}**	1.8157	-0.6607	2.1467	1.1156	-0.1195	1.9831	2.2047	0.2739	-0.1851
**χ_{bb}**	-4.2313	-1.4161	-2.0727	-3.3143	-0.8812	-4.6143	-4.6173	-2.5015	-1.2919
**χ_{cc}**	2.4155	2.0768	-0.0741	2.1987	1.0007	2.6312	2.4126	2.2276	1.4771
**ΔE(cm^{-1})**	1230	1333	1347	1369	1420	1455	1537	1675	1805
**ΔE$_{ZPE}$(cm^{-1})**	1005	1021	1043	1068	1122	1217	1231	1426	1529
**ΔG(cm^{-1})**	879	845	920	935	1007	1020	1075	1393	1376

A, B, and C are the rotational constants measured in MHz. χ_{aa}, χ_{bb} *and* χ_{cc} correspond to the quadrupole coupling constants in MHz. μ_a, μ_b,*and* μ_c represent the values of the three components of the dipole moment expressed in Debyes (note that 1D ≈ 3.33564•10^{-34} C · m). ΔE is the relative energy, ΔE$_{ZPE}$ represents the relative energy including the zero-point energy correction (ZPE), and ΔG is the Gibbs free energy at 1 atm and a temperature of 298 K.

Table S03 (Continuation). Spectroscopic and thermochemical parameters for the low-lying conformers of gabapentin computed at *MP2-GD3BJ/6-311++G(d,p)* and sorted by energy.

	**Distorted 2**	**Distorted 1**	**Distorted 3**
**A(MHz)**	1490.5	1417.3	1297.8
**B(MHz)**	687.3	703.2	711.2
**C(MHz)**	536.9	536.2	593.2
**μ_a(D)**	4.2	-0.6	-3.6
**μ_b(D)**	4.8	-0.8	5.6
**μ_c(d)**	0.4	-0.8	1.2
**χ_{aa}**	-1.6749	0.5026	-0.3134
**χ_{bb}**	0.4888	2.4407	-1.5511
**χ_{cc}**	1.1861	-2.9433	1.8645
**ΔE(cm^{-1})**	1607	1836	1989
**ΔE_{ZPE}(cm^{-1})**	1575	1626	1874
**ΔG(cm^{-1})**	1706	1532	1913

A, B, and C are the rotational constants measured in MHz. χ_{aa}, χ_{bb} *and* χ_{cc} correspond to the quadrupole coupling constants in MHz. μ_a, μ_b, *and* μ_c represent the values of the three components of the dipole moment expressed in Debyes (note that 1D ≈ $3.33564 \cdot 10^{-34}$ C · m). ΔE is the relative energy, ΔE_{ZPE} represents the relative energy including the zero-point energy correction (ZPE), and ΔG is the Gibbs free energy at 1 atm and a temperature of 298 K.

Table S04. Cartesian coordinates in Angstroms (Å) of the conformers of gabapentin from the optimized structures at B2PLYP-D3BJ/6-311++G(d,p).

Equatorial 1			
Atoms	x	y	z
C	0.739452	-0.440103	1.235701
C	1.839871	-1.348012	0.675433
C	2.954398	-0.53059	0.013349
C	2.394432	0.411042	-1.058074
C	1.292247	1.307949	-0.483966
C	0.146572	0.52567	0.191656
H	-0.055157	-1.044306	1.685233
H	1.162527	0.167063	2.046719
H	1.411323	-2.047043	-0.050563
H	2.251401	-1.960833	1.483162
H	3.70801	-1.196245	-0.418292
H	3.462699	0.066821	0.780857
H	3.194452	1.036417	-1.465974
H	2.009856	-0.176851	-1.89896
H	0.874288	1.965974	-1.249885
H	1.740534	1.958964	0.277738
C	-0.639996	-0.234131	-0.913807
H	0.008087	-0.945548	-1.421897
H	-0.991618	0.502444	-1.63779
C	-1.846739	-0.961457	-0.389948
O	-2.92472	-0.458607	-0.153836
C	-0.790603	1.512122	0.918945
H	-1.448675	0.947122	1.592445
H	-0.164632	2.150585	1.549101
H	-2.40734	1.905352	-0.268232
N	-1.539833	2.36486	-0.01061
H	-1.786726	3.241329	0.432351
O	-1.611944	-2.281864	-0.179449
H	-2.434686	-2.656426	0.168817

Equatorial 2			
Atoms	x	y	z
C	0.453937	0.194088	1.214123
C	1.470523	-0.95375	1.228681
C	2.778492	-0.534592	0.553472
C	2.521652	-0.054743	-0.877385
C	1.476496	1.066276	-0.912341
C	0.145389	0.712421	-0.206865
H	-0.466623	-0.12367	1.71289
H	0.860674	1.031009	1.798663
H	1.050288	-1.825668	0.718048
H	1.65666	-1.25744	2.262843
H	3.48924	-1.365845	0.550736
H	3.240497	0.27682	1.130533
H	3.448562	0.306479	-1.332727
H	2.190898	-0.902375	-1.486318
H	1.268882	1.357593	-1.948752
H	1.900839	1.949081	-0.417447
C	-0.603294	-0.344011	-1.074402
H	0.115771	-0.983094	-1.582038
H	-1.169633	0.182073	-1.851604
C	-1.552721	-1.308392	-0.365444
O	-1.366995	-2.504649	-0.364139
C	-0.664855	2.018852	-0.128021
H	-0.060342	2.776794	0.385235
H	-0.842444	2.378867	-1.145366
H	-1.8848	1.860243	1.529432
N	-1.981877	1.84693	0.518584
H	-2.598993	2.615359	0.278614
O	-2.625864	-0.770534	0.230725
H	-2.537173	0.219825	0.243563

Axial 1			
Atoms	x	y	z
C	-1.265772	0.588451	1.178577
C	-2.671123	0.594627	0.566619
C	-3.0254	-0.765221	-0.042304
C	-1.971201	-1.200688	-1.064169
C	-0.571488	-1.222332	-0.437894
C	-0.167412	0.121779	0.199732
H	-1.265851	-0.086428	2.044086
H	-1.002819	1.58505	1.543198
H	-3.398083	0.865981	1.338134
H	-2.742296	1.369268	-0.204809
H	-4.015366	-0.729499	-0.507118
H	-3.077827	-1.514473	0.757814
H	-1.990834	-0.52106	-1.923338
H	-2.20851	-2.195344	-1.453585
H	-0.549154	-1.995376	0.340069
H	0.171461	-1.517805	-1.186517
C	1.125047	-0.059784	1.041102
H	0.953891	-0.834599	1.789687
H	1.339966	0.882974	1.547186
C	2.335395	-0.415374	0.22188
O	2.999455	0.367039	-0.425084
C	0.061902	1.178855	-0.904586
H	0.681719	0.732853	-1.69363
H	-0.898462	1.420321	-1.362141
H	0.410095	3.201205	-0.972288
N	0.636836	2.418273	-0.371309
H	1.648652	2.340954	-0.351283
O	2.623682	-1.740294	0.256096
H	3.400782	-1.86778	-0.308147

Equatorial 3			
Atoms	x	y	z
C	1.244339	1.318866	-0.484633
C	2.395039	0.495187	-1.074591
C	3.028332	-0.402114	-0.007034
C	1.979106	-1.298041	0.658238
C	0.816986	-0.477486	1.22744
C	0.153745	0.459911	0.195629
H	1.65865	1.999752	0.269915
H	0.798001	1.945451	-1.26611
H	2.033248	-0.119012	-1.905928
H	3.14469	1.171879	-1.495493
H	3.824141	-1.010441	-0.446489
H	3.498633	0.230421	0.756574
H	1.599542	-2.026408	-0.065135
H	2.435859	-1.879391	1.464262
H	1.193047	0.141781	2.052581
H	0.061271	-1.145627	1.650936
C	-0.594442	-0.354989	-0.893745
H	-0.917108	0.32242	-1.689009
H	0.080036	-1.085284	-1.33514
C	-1.798334	-1.130025	-0.379137
O	-1.767736	-2.312319	-0.129561
O	-2.924514	-0.408421	-0.216187
H	-2.707125	0.546786	-0.338305
C	-0.814578	1.376505	0.968559
H	-1.410703	0.771602	1.656911
H	-0.219233	2.068561	1.5767
H	-2.360737	2.693841	0.627166
N	-1.765986	2.077929	0.081508
H	-1.273469	2.667213	-0.582358

Table S04 (Continuation).

Equatorial 4			
Atoms	x	y	z
C	0.799444	-0.504638	1.213029
C	1.919711	-1.357389	0.607585
C	2.997408	-0.485284	-0.044981
C	2.389872	0.471574	-1.075979
C	1.269434	1.314686	-0.457496
C	0.160197	0.474342	0.208777
H	0.034154	-1.150939	1.651749
H	1.217213	0.092187	2.034427
H	1.501638	-2.04834	-0.131991
H	2.361918	-1.981258	1.390039
H	3.7639	-1.112358	-0.510427
H	3.500073	0.10338	0.732976
H	3.162166	1.135178	-1.476947
H	2.009439	-0.101397	-1.929164
H	0.820728	1.983647	-1.195549
H	1.707496	1.955475	0.31889
C	-0.621443	-0.27577	-0.907867
H	0.04373	-0.960874	-1.430897
H	-0.997028	0.460197	-1.620014
C	-1.765347	-1.098867	-0.383606
O	-1.696399	-2.228436	0.041877
C	-0.795443	1.405264	0.981165
H	-1.465917	0.79091	1.599887
H	-0.187628	1.99727	1.67132
H	-2.375403	1.89415	-0.233985
N	-1.522115	2.327173	0.100991
H	-1.789457	3.164084	0.604178
O	-2.948304	-0.417006	-0.414439
H	-3.617586	-1.011497	-0.044687

Axial 2			
Atoms	x	y	z
C	-0.644908	-1.268482	-0.35806
C	-2.038331	-1.202731	-0.995779
C	-3.072025	-0.641152	-0.015492
C	-2.637179	0.727288	0.516991
C	-1.24163	0.66675	1.147401
C	-0.166279	0.084738	0.20598
H	-0.672186	-1.992316	0.465482
H	0.083824	-1.654929	-1.076751
H	-2.014519	-0.581767	-1.898267
H	-2.331019	-2.205147	-1.322382
H	-3.179022	-1.335915	0.827153
H	-4.053656	-0.571205	-0.494004
H	-3.352623	1.09005	1.261258
H	-2.64904	1.458642	-0.298822
H	-0.923843	1.66252	1.466226
H	-1.290696	0.034728	2.043309
C	1.111444	-0.121873	1.065411
H	0.891374	-0.858671	1.839756
H	1.375872	0.82373	1.541278
C	2.290451	-0.637208	0.28485
O	2.537005	-1.79465	0.040177
C	0.130263	1.059736	-0.954702
H	-0.799677	1.268258	-1.484954
H	0.789753	0.553142	-1.674459
H	1.680547	2.270633	-0.370544
N	0.675321	2.337316	-0.484019
H	0.499708	3.070184	-1.160324
O	3.090841	0.375426	-0.16356
H	3.804355	-0.041752	-0.668362

Equatorial 5			
Atoms	x	y	z
C	-0.573287	-0.3805	1.266729
C	-1.56597	-1.441419	0.779543
C	-2.792971	-0.785836	0.138751
C	-2.382577	0.152567	-0.999743
C	-1.356815	1.191976	-0.531106
C	-0.114774	0.585547	0.157336
H	-1.055658	0.214174	2.053099
H	0.297284	-0.853553	1.734017
H	-1.079167	-2.104151	0.05666
H	-1.870192	-2.07043	1.621655
H	-3.331719	-0.210657	0.902548
H	-3.485556	-1.548634	-0.229442
H	-1.971066	-0.438477	-1.825085
H	-3.260012	0.667748	-1.402617
H	-1.844852	1.860046	0.191878
H	-1.037965	1.8126	-1.374261
C	0.748344	-0.128796	-0.918453
H	0.132228	-0.751733	-1.563686
H	1.219406	0.654054	-1.517638
C	1.843338	-0.977215	-0.332302
O	2.814943	-0.576175	0.26938
O	1.629621	-2.306306	-0.535401
H	2.375924	-2.768157	-0.125556
C	0.711756	1.715919	0.804144
H	0.103477	2.1559	1.607178
H	1.598977	1.280616	1.26627
H	0.456911	3.428296	-0.324846
N	1.155849	2.709808	-0.180196
H	2.002266	3.170084	0.129976

Axial 3			
Atoms	x	y	z
C	0.493884	-1.242636	-0.463054
C	1.940876	-1.359477	-0.962226
C	2.9501	-0.978858	0.124784
C	2.650046	0.411623	0.69317
C	1.210894	0.486556	1.21271
C	0.151239	0.135609	0.143809
H	-0.201948	-1.468996	-1.274676
H	0.332985	-2.002219	0.313726
H	2.121357	-2.383972	-1.301784
H	2.08956	-0.717562	-1.836816
H	3.969553	-1.019915	-0.27066
H	2.896164	-1.712874	0.938747
H	3.34428	0.647167	1.505555
H	2.815418	1.167583	-0.083365
H	0.996129	1.474822	1.631873
H	1.108109	-0.224685	2.042101
C	-1.205615	0.084363	0.867868
H	-1.137164	-0.548708	1.756375
H	-1.455195	1.092914	1.215618
C	-2.393128	-0.386535	0.067283
O	-2.513061	-0.413371	-1.136938
O	-3.401968	-0.780357	0.890765
H	-4.142828	-1.023317	0.316839
C	0.116154	1.212802	-0.962978
H	1.06312	1.199053	-1.513242
H	-0.669577	0.945125	-1.668735
H	-0.562994	3.144052	-1.146412
N	-0.195695	2.543441	-0.418024
H	0.636439	2.996589	-0.057789

Table S04 (Continuation).

Equatorial 6			
Atoms	x	y	z
C	-1.291727	1.298202	-0.476585
C	-2.395655	0.409779	-1.062534
C	-2.959705	-0.541186	-0.002229
C	-1.847476	-1.368697	0.650004
C	-0.746948	-0.468333	1.221216
C	-0.145119	0.510573	0.193021
H	-0.888042	1.956305	-1.254014
H	-1.738043	1.948591	0.286415
H	-2.008172	-0.170512	-1.907271
H	-3.192617	1.041303	-1.466643
H	-3.714805	-1.198298	-0.4438
H	-3.467036	0.048798	0.771416
H	-2.260392	-1.989649	1.45065
H	-1.420709	-2.060654	-0.083859
H	0.045624	-1.078068	1.665286
H	-1.171329	0.130684	2.037236
C	0.644037	-0.236709	-0.91776
H	0.977934	0.493241	-1.657279
H	0.002315	-0.961573	-1.416137
C	1.872845	-0.940478	-0.410375
O	2.962086	-0.431985	-0.259837
C	0.782599	1.488902	0.960415
H	0.148965	2.055529	1.651425
H	1.47266	0.91018	1.580617
H	2.320403	1.958658	-0.313499
N	1.584848	2.434273	0.195666
H	1.028524	2.976874	-0.453588
O	1.640136	-2.244097	-0.111156
H	2.475911	-2.604986	0.220296

Equatorial 8			
Atoms	x	y	z
C	0.578432	-0.393314	1.25832
C	1.598318	-1.4255	0.76635
C	2.815528	-0.732842	0.146547
C	2.393978	0.209256	-0.984658
C	1.337025	1.217201	-0.516113
C	0.103701	0.572652	0.155819
H	1.040734	0.204713	2.054269
H	-0.282674	-0.894712	1.713433
H	1.131129	-2.089218	0.031996
H	1.908718	-2.058072	1.6033
H	3.333771	-0.154765	0.922392
H	3.529134	-1.474308	-0.224806
H	3.261851	0.752107	-1.371597
H	2.007381	-0.382227	-1.821501
H	1.801001	1.891405	0.216826
H	1.011065	1.83617	-1.35811
C	-0.728444	-0.15202	-0.939914
H	-1.267477	0.618796	-1.495995
H	-0.075647	-0.693898	-1.62103
C	-1.708747	-1.164272	-0.415125
O	-1.594219	-2.366867	-0.493962
C	-0.746628	1.679681	0.812337
H	-1.621905	1.224172	1.277564
H	-0.144216	2.127053	1.615978
H	-2.04404	3.146168	0.180972
N	-1.219114	2.670047	-0.162203
H	-0.516761	3.378301	-0.339255
O	-2.787071	-0.591255	0.188944
H	-3.346928	-1.322079	0.489735

Equatorial 7			
Atoms	x	y	z
C	0.818708	-0.54055	1.195859
C	1.944099	-1.375407	0.575745
C	3.011229	-0.484894	-0.068196
C	2.389742	0.477476	-1.084942
C	1.265865	1.304307	-0.448909
C	0.163554	0.450887	0.212632
H	0.058264	-1.19844	1.624529
H	1.234178	0.045888	2.025517
H	1.531887	-2.061003	-0.172331
H	2.394386	-2.005185	1.348651
H	3.782042	-1.096843	-0.546068
H	3.511027	0.098877	0.715056
H	3.153256	1.151444	-1.485126
H	2.004819	-0.089731	-1.939865
H	0.823717	1.971755	-1.196677
H	1.702298	1.945068	0.328291
C	-0.62104	-0.289854	-0.906709
H	0.031421	-1.003121	-1.408874
H	-0.965919	0.43702	-1.643901
C	-1.800826	-1.065606	-0.386648
O	-1.76336	-2.160564	0.124308
C	-0.787768	1.363242	1.028438
H	-1.479078	0.732811	1.59713
H	-0.17716	1.888753	1.770126
H	-1.011608	2.979704	-0.236004
N	-1.585428	2.355903	0.318008
H	-2.26931	1.918157	-0.287058
O	-2.96916	-0.378516	-0.533149
H	-3.667443	-0.931437	-0.152347

Equatorial 9			
Atoms	x	y	z
C	-0.725838	-0.001288	1.230849
C	-1.743395	-1.129971	1.037334
C	-2.953115	-0.65917	0.22477
C	-2.5187	-0.058806	-1.115764
C	-1.483954	1.054774	-0.917172
C	-0.247915	0.624682	-0.096336
H	-1.197018	0.798265	1.819182
H	0.125298	-0.374388	1.801627
H	-1.262484	-1.976953	0.536605
H	-2.06599	-1.497617	2.015957
H	-3.494802	0.103279	0.798894
H	-3.650902	-1.486019	0.06147
H	-2.107293	-0.848693	-1.753745
H	-3.38471	0.343609	-1.650198
H	-1.158988	1.44876	-1.887204
H	-1.96662	1.886015	-0.387781
C	0.589544	-0.353538	-0.957677
H	-0.025676	-1.21177	-1.23789
H	0.884471	0.152827	-1.878836
C	1.819979	-0.935996	-0.305394
O	1.832918	-1.632118	0.685383
O	2.941839	-0.686169	-1.026741
H	3.669456	-1.102998	-0.54355
C	0.58357	1.886335	0.18961
H	0.880963	2.332011	-0.765239
H	-0.065849	2.615007	0.697944
H	1.604537	1.376133	1.906119
N	1.807269	1.580032	0.934072
H	2.44561	2.366475	0.917687

Table S04 (Continuation).

Axial 4			
Atoms	x	y	z
C	-0.675006	-1.253195	-0.408402
C	-2.057079	-1.126922	-1.059848
C	-3.088538	-0.59905	-0.058117
C	-2.636844	0.730478	0.554498
C	-1.241561	0.612635	1.179689
C	-0.16569	0.064747	0.213371
H	-0.73384	-2.001855	0.390231
H	0.059972	-1.630387	-1.1256
H	-2.012071	-0.460665	-1.928608
H	-2.366699	-2.104644	-1.439875
H	-3.215669	-1.336887	0.74372
H	-4.064713	-0.4823	-0.537754
H	-3.347856	1.056449	1.319501
H	-2.642348	1.50791	-0.217786
H	-0.926065	1.581272	1.58652
H	-1.297733	-0.067649	2.037896
C	1.105659	-0.222854	1.053972
H	1.361837	0.663002	1.641919
H	0.880981	-1.033345	1.748024
C	2.324477	-0.651574	0.247845
O	2.684576	-1.800885	0.150474
C	0.12781	1.075152	-0.915301
H	0.686817	0.569784	-1.707136
H	-0.811145	1.419303	-1.356649
H	1.092256	2.872526	-1.203816
N	0.965985	2.202515	-0.451421
H	0.509818	2.703285	0.304874
O	2.994136	0.346908	-0.360505
H	2.475503	1.18192	-0.261507

Axial 5			
Atoms	x	y	z
C	0.35358	-1.179393	-0.51792
C	1.763262	-1.351182	-1.101293
C	2.846436	-1.087957	-0.050912
C	2.659129	0.284719	0.602925
C	1.257488	0.409706	1.209029
C	0.128856	0.176873	0.183126
H	-0.391946	-1.313763	-1.30985
H	0.176338	-1.976885	0.214132
H	1.865036	-2.364171	-1.502314
H	1.906488	-0.671306	-1.947557
H	3.839809	-1.166096	-0.502904
H	2.790061	-1.860591	0.726188
H	3.409826	0.438375	1.383999
H	2.824096	1.070746	-0.143454
H	1.118789	1.384963	1.685053
H	1.158567	-0.340098	2.003691
C	-1.207679	0.162115	0.970237
H	-1.412759	1.185158	1.293904
H	-1.109647	-0.482422	1.844491
C	-2.380414	-0.304949	0.150895
O	-2.882376	0.285045	-0.780788
O	-2.843353	-1.517005	0.55902
H	-3.585592	-1.735062	-0.023923
C	0.089082	1.313713	-0.861889
H	-0.778579	1.154674	-1.503157
H	0.976505	1.25265	-1.500466
H	-0.516624	3.279577	-0.853615
N	-0.053193	2.632248	-0.227933
H	0.849363	3.032315	0.000264

Axial 6			
Atoms	x	y	z
C	-0.417069	-0.352775	1.037432
C	-1.81669	0.015421	1.543589
C	-2.908627	-0.634813	0.687355
C	-2.739962	-0.278562	-0.793508
C	-1.328515	-0.618593	-1.29089
C	-0.185001	0.004135	-0.448509
H	0.350296	0.108907	1.665181
H	-0.27336	-1.433614	1.147471
H	-1.918222	-0.296202	2.58717
H	-1.949714	1.104803	1.536685
H	-3.900382	-0.336355	1.040025
H	-2.847726	-1.724109	0.803382
H	-3.472814	-0.820956	-1.398497
H	-2.955356	0.78538	-0.941029
H	-1.211598	-0.324614	-2.340442
H	-1.207882	-1.706803	-1.260935
C	1.149477	-0.619157	-0.952913
H	0.967858	-1.656511	-1.232775
H	1.471961	-0.095696	-1.860981
C	2.346429	-0.68025	0.000555
O	2.888887	-1.726783	0.263103
O	2.796293	0.477388	0.497913
H	2.165964	1.221673	0.281417
C	-0.203922	1.529519	-0.687609
H	0.021847	1.707963	-1.742324
H	-1.20607	1.926823	-0.506197
H	0.487289	2.382603	1.065196
N	0.807403	2.254441	0.109527
H	0.967924	3.181233	-0.271174

Axial 7			
Atoms	x	y	z
C	-1.260016	0.609488	1.164546
C	-2.662158	0.626443	0.545674
C	-3.03159	-0.745963	-0.024709
C	-1.97971	-1.225485	-1.028728
C	-0.5794	-1.238472	-0.402258
C	-0.159797	0.120526	0.197725
H	-1.271757	-0.055952	2.037182
H	-0.999877	1.603783	1.545276
H	-3.388556	0.929473	1.305764
H	-2.715557	1.378771	-0.248555
H	-4.018987	-0.709724	-0.494347
H	-3.098077	-1.46938	0.79781
H	-2.224854	-2.231993	-1.380759
H	-1.994444	-0.578592	-1.912011
H	-0.562185	-1.990854	0.396119
H	0.160341	-1.560441	-1.142237
C	1.127244	-0.054934	1.048171
H	0.951928	-0.829239	1.796976
H	1.344481	0.883555	1.561781
C	2.348308	-0.406473	0.241621
O	3.057251	0.387824	-0.337255
C	0.074556	1.141466	-0.949509
H	0.73985	0.687334	-1.688731
H	-0.873409	1.312118	-1.46293
H	0.108275	2.916809	0.11171
N	0.637784	2.442629	-0.60963
H	1.601127	2.353437	-0.308653
O	2.585298	-1.741572	0.20521
H	3.372198	-1.8671	-0.345835

Table S04 (Continuation).

Axial 8			
Atoms	x	y	z
C	-0.589554	-0.020678	-1.190852
C	-2.074145	-0.398359	-1.253225
C	-2.390029	-1.51998	-0.25824
C	-1.995697	-1.121917	1.167573
C	-0.521656	-0.698384	1.243411
C	-0.133493	0.396025	0.224528
H	-0.36005	0.781958	-1.899533
H	-0.001577	-0.88709	-1.500515
H	-2.325285	-0.714915	-2.270092
H	-2.706437	0.471205	-1.035628
H	-1.829892	-2.417039	-0.548383
H	-3.452248	-1.780085	-0.298521
H	-2.647636	-0.31172	1.511329
H	-2.163007	-1.959389	1.851792
H	0.098894	-1.57815	1.055198
H	-0.28267	-0.348234	2.255441
C	1.382758	0.649967	0.258555
H	1.681612	0.979246	1.260653
H	1.623769	1.487892	-0.402474
C	2.300442	-0.49063	-0.103053
O	2.006022	-1.600872	-0.483569
C	-0.801304	1.727339	0.641305
H	-1.875206	1.570033	0.786316
H	-0.393231	2.018714	1.614408
H	-1.103942	2.742723	-1.125663
N	-0.513142	2.814839	-0.304856
H	-0.714013	3.712952	0.119507
O	3.599312	-0.115557	0.055494
H	4.136308	-0.880835	-0.195253

Axial 9			
Atoms	x	y	z
C	0.534793	-0.695004	-0.900429
C	1.919417	-0.342878	-1.453213
C	3.017977	-0.601437	-0.418511
C	2.740502	0.164486	0.878043
C	1.343671	-0.153687	1.425969
C	0.200952	0.053188	0.403425
H	-0.228949	-0.478557	-1.647854
H	0.496968	-1.772774	-0.698657
H	2.106413	-0.930304	-2.35714
H	1.939587	0.708671	-1.760218
H	3.997891	-0.32488	-0.819441
H	3.057084	-1.676065	-0.19952
H	2.843019	1.239313	0.694993
H	3.487641	-0.08733	1.63704
H	1.142979	0.446968	2.321301
H	1.330002	-1.202943	1.745445
C	-1.070923	-0.545717	1.065634
H	-1.262523	-0.013842	2.000568
H	-0.876802	-1.596355	1.292539
C	-2.337883	-0.466863	0.254289
O	-3.147912	0.43482	0.295876
O	-2.506897	-1.545167	-0.547335
H	-3.326368	-1.395376	-1.041481
C	0.027801	1.573227	0.16389
H	-0.297195	2.025502	1.112883
H	1.007103	1.997279	-0.061292
H	-0.631312	2.816604	-1.327812
N	-0.854243	1.899962	-0.960394
H	-1.825203	1.915248	-0.671505

Equatorial 10			
Atoms	x	y	z
C	-0.638286	0.001864	1.224236
C	-1.672344	-1.115526	1.055508
C	-2.911403	-0.620175	0.302564
C	-2.530384	0.001557	-1.044882
C	-1.475664	1.100824	-0.871302
C	-0.210673	0.644009	-0.108859
H	-1.077973	0.797534	1.840258
H	0.23795	-0.353518	1.765408
H	-1.95906	-1.494292	2.041279
H	-1.22523	-1.961391	0.522544
H	-3.420267	0.136842	0.912694
H	-3.624076	-1.437286	0.154767
H	-2.15894	-0.779969	-1.716844
H	-3.41483	0.424188	-1.53143
H	-1.926512	1.924097	-0.302986
H	-1.191147	1.513818	-1.846025
C	0.55664	-0.343335	-1.03407
H	-0.089495	-1.184175	-1.287675
H	0.81471	0.181791	-1.956207
C	1.840676	-0.910533	-0.488104
O	2.955018	-0.505635	-0.740818
O	1.631436	-1.979252	0.318608
H	2.50261	-2.259487	0.635489
C	0.642744	1.901993	0.160017
H	0.909305	2.353148	-0.807711
H	-0.001368	2.619551	0.67688
H	2.05635	2.48878	1.528651
N	1.807583	1.65275	1.015373
H	2.615427	1.376145	0.470327

Equatorial 11			
Atoms	x	y	z
C	0.709376	-0.024207	1.226371
C	1.766035	-1.11896	1.042756
C	2.968087	-0.613162	0.239069
C	2.528316	-0.015132	-1.101138
C	1.471565	1.075667	-0.894651
C	0.235011	0.599402	-0.102216
H	-0.144722	-0.41953	1.777483
H	1.145524	0.78297	1.831928
H	1.320444	-1.983496	0.538957
H	2.092499	-1.47477	2.02465
H	3.4865	0.160041	0.820249
H	3.687476	-1.421582	0.077412
H	3.389316	0.407619	-1.627738
H	2.133561	-0.807777	-1.746181
H	1.153607	1.488755	-1.85882
H	1.931905	1.901038	-0.336852
C	-0.54881	-0.402997	-0.990171
H	0.035473	-1.315458	-1.123981
H	-0.695354	0.047845	-1.973568
C	-1.918898	-0.818553	-0.512283
O	-2.964836	-0.509777	-1.034839
C	-0.650034	1.826687	0.183748
H	-0.010247	2.625872	0.584358
H	-1.060718	2.189731	-0.763318
H	-1.50664	1.574816	2.043997
N	-1.771487	1.498388	1.06961
H	-2.555871	2.119334	0.915043
O	-1.858914	-1.683162	0.535164
H	-2.776219	-1.873085	0.777595

Table S04 (Continuation).

Equatorial 12			
Atoms	x	y	z
C	-1.498952	0.553778	0.98178
C	-2.208652	-0.803598	1.060768
C	-2.427427	-1.392515	-0.33615
C	-1.107045	-1.487362	-1.107014
C	-0.416861	-0.121217	-1.18784
C	-0.174123	0.526081	0.18909
H	-2.173332	1.25521	0.479413
H	-1.313521	0.948603	1.988488
H	-1.62647	-1.507491	1.665728
H	-3.166513	-0.680289	1.575525
H	-3.120905	-0.748544	-0.891121
H	-2.89854	-2.377394	-0.262374
H	-0.44802	-2.216166	-0.623158
H	-1.289347	-1.861251	-2.119183
H	-1.03992	0.570116	-1.762194
H	0.531588	-0.205688	-1.730356
C	0.872879	-0.267516	1.020804
H	1.119484	0.313282	1.913788
H	0.463096	-1.224874	1.336811
C	2.160705	-0.518861	0.280699
O	3.024373	0.299148	0.056866
O	2.26424	-1.806279	-0.143277
H	3.105999	-1.872037	-0.618013
C	0.347656	1.969658	0.011787
H	0.605794	2.364421	1.005653
H	1.275807	1.939686	-0.560953
H	-0.117976	3.58972	-1.160773
N	-0.603585	2.825749	-0.707488
H	-1.280108	3.238823	-0.07711

Axial 11			
Atoms	x	y	z
C	0.651213	0.017505	1.205657
C	2.064852	-0.575552	1.17735
C	2.136673	-1.775898	0.227957
C	1.673986	-1.395532	-1.181916
C	0.272907	-0.769194	-1.157095
C	0.142518	0.428486	-0.191684
H	-0.033655	-0.725608	1.6223
H	0.601284	0.877784	1.880727
H	2.79532	0.180314	0.864561
H	2.350017	-0.878515	2.189229
H	1.488738	-2.571995	0.615134
H	3.15275	-2.180796	0.197504
H	1.660346	-2.280006	-1.826099
H	2.394957	-0.70302	-1.628716
H	-0.027741	-0.461574	-2.165322
H	-0.436715	-1.545396	-0.848197
C	-1.335019	0.898558	-0.120224
H	-1.627362	1.307049	-1.089512
H	-1.407262	1.694903	0.621812
C	-2.305869	-0.186934	0.257369
O	-2.426603	-0.693263	1.35065
C	0.953448	1.620202	-0.745736
H	1.987104	1.305953	-0.927169
H	0.532081	1.891962	-1.718474
H	1.479503	2.703668	0.925348
N	0.866346	2.800755	0.123737
H	1.168743	3.629056	-0.375647
O	-3.07445	-0.57546	-0.796943
H	-3.662354	-1.269136	-0.463351

Axial 10			
Atoms	x	y	z
C	-0.363871	0.036234	1.140572
C	-1.810593	-0.366381	1.446455
C	-2.231108	-1.560956	0.584328
C	-2.05202	-1.257173	-0.906346
C	-0.622135	-0.794884	-1.222798
C	-0.137119	0.379141	-0.345994
H	0.280543	-0.809202	1.407212
H	-0.040069	0.871343	1.768635
H	-1.903199	-0.614085	2.508167
H	-2.490832	0.474481	1.26471
H	-1.61076	-2.425038	0.852603
H	-3.268723	-1.838586	0.792985
H	-2.777519	-0.494494	-1.208861
H	-2.279385	-2.146986	-1.501095
H	0.061965	-1.636779	-1.083214
H	-0.544579	-0.508948	-2.278667
C	1.36481	0.667079	-0.613926
H	1.619051	1.625969	-0.163389
H	1.517854	0.725604	-1.695096
C	2.289184	-0.399871	-0.093473
O	2.480872	-1.484946	-0.596887
C	-0.902925	1.661048	-0.74386
H	-0.621788	1.91213	-1.771406
H	-1.978457	1.453809	-0.754853
H	-1.040284	2.756797	0.995762
N	-0.556681	2.807236	0.106095
H	-0.84527	3.673021	-0.33433
O	2.912031	-0.025396	1.055604
H	3.469645	-0.772376	1.318688

Axial 12			
Atoms	x	y	z
C	0.649348	0.978499	-0.623137
C	2.023579	1.434062	-0.120582
C	3.09824	0.388687	-0.433156
C	2.724784	-0.975856	0.153274
C	1.327928	-1.420053	-0.300903
C	0.214596	-0.380337	-0.037111
H	-0.114064	1.734136	-0.430864
H	0.693081	0.876766	-1.714434
H	2.280328	2.3909	-0.584756
H	1.994203	1.614263	0.961062
H	4.072307	0.710833	-0.052833
H	3.197192	0.297941	-1.522125
H	3.456927	-1.731159	-0.148558
H	2.770207	-0.925942	1.246122
H	1.059934	-2.368786	0.17906
H	1.359741	-1.615419	-1.379914
C	-1.058063	-0.899257	-0.750479
H	-1.282601	-1.9067	-0.393613
H	-0.848351	-0.956248	-1.822511
C	-2.291613	-0.039437	-0.605502
O	-2.458643	1.043632	-1.117638
O	-3.262711	-0.661393	0.11183
H	-3.996485	-0.033018	0.169642
C	-0.044933	-0.292975	1.479738
H	-0.455925	-1.253102	1.807142
H	0.905499	-0.15959	2.009439
H	-0.614953	1.668816	1.752993
N	-1.028521	0.744697	1.806382
H	-1.369759	0.625513	2.753097

Table S04 (Continuation).

Equatorial 13			
Atoms	x	y	z
C	-0.636233	-0.107274	-1.201853
C	-1.553298	-1.327099	-1.060754
C	-2.744972	-1.019278	-0.148596
C	-2.279958	-0.504087	1.217322
C	-1.348845	0.705085	1.069377
C	-0.137926	0.443497	0.149974
H	0.213179	-0.34706	-1.846659
H	-1.208756	0.688393	-1.691528
H	-0.98902	-2.178045	-0.664238
H	-1.904606	-1.630956	-2.051379
H	-3.371859	-1.907871	-0.026402
H	-3.369393	-0.252961	-0.624517
H	-3.143191	-0.222085	1.827966
H	-1.775631	-1.312861	1.759029
H	-1.906117	1.543314	0.642504
H	-0.991723	1.03234	2.053285
C	0.809908	-0.555701	0.870917
H	0.304467	-1.512619	1.003029
H	1.055345	-0.160815	1.858577
C	2.086576	-0.844449	0.125768
O	2.184837	-1.502785	-0.885112
C	0.641691	1.757247	-0.068302
H	0.968568	2.127883	0.908356
H	1.55143	1.53875	-0.645285
H	-0.214452	2.683464	-1.698084
N	-0.177077	2.803555	-0.692699
H	0.228382	3.715814	-0.521363
O	3.169598	-0.272963	0.71845
H	3.935777	-0.511235	0.175773

Axial 13			
Atoms	x	y	z
C	0.640857	0.895926	-0.742082
C	2.007742	1.413718	-0.281428
C	3.090016	0.346938	-0.468496
C	2.719131	-0.941581	0.270621
C	1.328233	-1.446846	-0.135677
C	0.207174	-0.390092	-0.011936
H	-0.128914	1.66066	-0.629205
H	0.697721	0.674434	-1.815099
H	2.263511	2.316901	-0.843526
H	1.9679	1.70843	0.774734
H	4.060421	0.715913	-0.12292
H	3.19493	0.132605	-1.539447
H	3.458103	-1.72261	0.067014
H	2.75528	-0.761201	1.350129
H	1.061039	-2.328283	0.459316
H	1.368791	-1.778749	-1.180719
C	-1.052342	-1.000921	-0.679043
H	-1.193135	-2.014884	-0.299115
H	-0.883787	-1.056674	-1.75821
C	-2.355972	-0.278543	-0.438942
O	-3.229472	-0.649054	0.311904
O	-2.488952	0.82232	-1.221687
H	-3.328681	1.231631	-0.969177
C	-0.085994	-0.124772	1.480977
H	-0.587063	-1.00761	1.888139
H	0.855774	-0.017375	2.029785
H	-0.449949	1.89037	1.735804
N	-0.976477	1.027745	1.6649
H	-1.522277	0.933139	2.512491

Equatorial 14			
Atoms	x	y	z
C	0.558491	0.059342	1.202713
C	1.062572	-1.384461	1.308699
C	2.316417	-1.586438	0.452455
C	2.056472	-1.197991	-1.006328
C	1.49593	0.225483	-1.121391
C	0.248501	0.486133	-0.247297
H	1.348749	0.717798	1.57861
H	-0.319782	0.212332	1.83386
H	1.277862	-1.61905	2.355551
H	0.282235	-2.087115	0.991848
H	2.661073	-2.623318	0.513337
H	3.122089	-0.958849	0.852892
H	2.981866	-1.268415	-1.586275
H	1.364479	-1.921206	-1.453535
H	2.260716	0.943597	-0.812726
H	1.255467	0.449425	-2.168271
C	-0.930179	-0.308424	-0.844939
H	-0.650742	-1.343263	-1.046042
H	-1.189848	0.113561	-1.824499
C	-2.217443	-0.354259	-0.055442
O	-2.553961	0.358464	0.861559
C	-0.100715	1.990011	-0.308011
H	-0.204215	2.263657	-1.363725
H	-1.072328	2.148098	0.170114
H	0.841622	2.918467	1.270088
N	0.957673	2.834968	0.266685
H	0.897433	3.773523	-0.109773
O	-3.028112	-1.338996	-0.529201
H	-3.84408	-1.29615	-0.009864

Equatorial 15			
Atoms	x	y	z
C	0.425719	-0.079997	-1.168703
C	1.110918	-1.450118	-1.113818
C	2.435296	-1.36189	-0.349588
C	2.219244	-0.800401	1.058817
C	1.496006	0.551207	1.019783
C	0.175476	0.537854	0.221159
H	1.078774	0.603738	-1.721021
H	-0.514562	-0.142241	-1.728917
H	0.452325	-2.182435	-0.636097
H	1.28574	-1.809293	-2.132668
H	3.121749	-0.704233	-0.897124
H	2.911626	-2.345626	-0.297059
H	1.64879	-1.525174	1.651247
H	3.179281	-0.679909	1.570111
H	2.145401	1.296728	0.551942
H	1.294272	0.900923	2.039684
C	-0.878204	-0.271845	1.031186
H	-0.456209	-1.220593	1.357928
H	-1.148778	0.30757	1.917817
C	-2.150479	-0.550781	0.273744
O	-3.047648	0.235733	0.069757
O	-2.19725	-1.827319	-0.19299
H	-3.033823	-1.912897	-0.673614
C	-0.344526	1.985079	0.074725
H	-1.35678	1.963939	-0.342059
H	-0.424977	2.412109	1.079604
H	0.453303	3.805597	-0.441459
N	0.573563	2.833927	-0.700665
H	0.371422	2.772347	-1.691632

Table S04 (Continuation).

Equatorial 16			
Atoms	x	y	z
C	0.690342	-0.015157	1.225537
C	1.710491	-1.14079	1.026259
C	2.933087	-0.651221	0.244498
C	2.520618	-0.026387	-1.091707
C	1.477245	1.079006	-0.891366
C	0.229698	0.637621	-0.092412
H	-0.165159	-0.395054	1.787182
H	1.152	0.773264	1.834964
H	1.238707	-1.977635	0.50083
H	2.01721	-1.527613	2.002507
H	3.635574	-1.47325	0.077706
H	3.462579	0.101096	0.842626
H	3.394102	0.390772	-1.602021
H	2.127289	-0.806338	-1.753265
H	1.165327	1.487972	-1.859506
H	1.948014	1.904279	-0.342748
C	-0.584472	-0.33336	-0.991621
H	0.062401	-1.149255	-1.318949
H	-0.918592	0.206207	-1.88001
C	-1.769073	-1.002748	-0.344731
O	-1.722963	-1.841239	0.525489
C	-0.593644	1.915847	0.204739
H	0.069385	2.614045	0.727027
H	-0.856077	2.390619	-0.746863
H	-2.551002	1.323005	0.47713
N	-1.818929	1.798734	0.987532
H	-1.666568	1.31243	1.862558
O	-2.954664	-0.588856	-0.878159
H	-3.64742	-1.101786	-0.435701

Equatorial 18			
Atoms	x	y	z
C	0.570007	0.045672	1.210371
C	1.590696	-1.092315	1.095907
C	2.857749	-0.62973	0.370724
C	2.521004	-0.047853	-1.005134
C	1.481736	1.07338	-0.892326
C	0.188746	0.675485	-0.142281
H	-0.323303	-0.30811	1.732607
H	1.000312	0.84164	1.833017
H	1.144512	-1.937733	0.563029
H	1.840617	-1.456241	2.097114
H	3.564105	-1.459109	0.270158
H	3.356516	0.139964	0.973159
H	3.423371	0.344728	-1.483607
H	2.154418	-0.84726	-1.65857
H	1.222939	1.455819	-1.886483
H	1.93695	1.909531	-0.347482
C	-0.594066	-0.308419	-1.061281
H	0.071979	-1.102401	-1.398922
H	-0.939153	0.24581	-1.936798
C	-1.805694	-0.968766	-0.456163
O	-2.944049	-0.556272	-0.501966
C	-0.616531	1.985252	0.082665
H	0.070491	2.706551	0.538316
H	-0.885849	2.392169	-0.898236
H	-2.540309	1.369338	0.493982
N	-1.823955	1.960123	0.897053
H	-1.636468	1.639686	1.839016
O	-1.497003	-2.138863	0.159567
H	-2.326901	-2.49039	0.514618

Equatorial 17			
Atoms	x	y	z
C	1.478448	1.093962	-0.885424
C	2.527225	-0.010398	-1.064919
C	2.919445	-0.63416	0.278228
C	1.685156	-1.122269	1.043092
C	0.666121	0.005589	1.228884
C	0.224277	0.65116	-0.097752
H	1.940406	1.923694	-0.335542
H	1.179305	1.495448	-1.860887
H	2.146231	-0.791959	-1.731616
H	3.407859	0.407745	-1.562258
H	3.440318	0.119058	0.883075
H	3.625223	-1.455494	0.121044
H	1.978982	-1.508625	2.023624
H	1.219498	-1.959182	0.512721
H	1.125988	0.796972	1.835897
H	-0.205003	-0.343511	1.782479
C	-0.577424	-0.326606	-1.003709
H	-0.940757	0.22167	-1.875979
H	0.08822	-1.115425	-1.355792
C	-1.730803	-1.045173	-0.351037
O	-1.640556	-1.972316	0.417701
O	-2.942762	-0.568813	-0.76261
H	-3.609435	-1.100408	-0.303011
C	-0.609498	1.916391	0.183734
H	0.021569	2.598441	0.760867
H	-0.821003	2.413428	-0.775275
H	-2.604513	1.424893	0.368196
N	-1.820638	1.652365	0.967487
H	-2.079557	2.46476	1.51273

Equatorial 19			
Atoms	x	y	z
C	-0.631707	-0.136938	-1.210048
C	-1.551338	-1.351896	-1.043149
C	-2.741158	-1.030431	-0.133196
C	-2.276462	-0.481866	1.219774
C	-1.352001	0.728077	1.035956
C	-0.137421	0.447815	0.127559
H	0.220228	-0.395302	-1.843421
H	-1.196117	0.646356	-1.730404
H	-0.985362	-2.194822	-0.632641
H	-1.905873	-1.674836	-2.026421
H	-3.362614	-1.919226	0.011201
H	-3.37333	-0.279208	-0.623524
H	-3.139152	-0.190386	1.826537
H	-1.762935	-1.271397	1.77945
H	-1.005577	1.098431	2.008137
H	-1.940691	1.533607	0.582028
C	0.80155	-0.539041	0.877349
H	0.297004	-1.493796	1.027563
H	1.043556	-0.122701	1.857068
C	2.081408	-0.843233	0.142367
O	2.180059	-1.526829	-0.851657
C	0.665138	1.755266	-0.112992
H	1.068685	2.093146	0.847514
H	1.528167	1.52281	-0.744853
H	-0.366314	2.664116	-1.649676
N	-0.016798	2.890165	-0.726493
H	-0.798302	3.208844	-0.167111
O	3.159898	-0.253908	0.721396
H	3.928504	-0.497419	0.18438

Table S04 (Continuation).

Axial 14			
Atoms	x	y	z
C	0.692611	0.01122	1.233145
C	2.102526	-0.588381	1.16758
C	2.175148	-1.752518	0.174681
C	1.691651	-1.324255	-1.214261
C	0.276099	-0.738234	-1.143149
C	0.149862	0.432836	-0.147029
H	0.012051	-0.727589	1.6658
H	0.682407	0.883648	1.891151
H	2.3943	-0.926689	2.166307
H	2.829467	0.180633	0.882247
H	3.195177	-2.144838	0.11814
H	1.539627	-2.570674	0.535922
H	2.388427	-0.5924	-1.637557
H	1.693715	-2.179186	-1.897289
H	-0.060333	-0.417608	-2.13579
H	-0.401738	-1.541124	-0.831372
C	-1.335023	0.875733	-0.028155
H	-1.644742	1.364572	-0.955324
H	-1.421197	1.585803	0.797369
C	-2.288713	-0.254708	0.255762
O	-2.416576	-0.834054	1.310036
C	0.926255	1.650978	-0.696515
H	1.987898	1.407553	-0.745082
H	0.593933	1.826533	-1.731034
H	1.544547	3.487462	-0.00961
N	0.788614	2.83505	0.159311
H	-0.073895	3.331259	-0.03247
O	-3.024151	-0.586945	-0.839726
H	-3.596623	-1.320741	-0.571415

Equatorial 20			
Atoms	x	y	z
C	0.415426	-0.081025	1.175133
C	1.084699	-1.459329	1.118663
C	2.411789	-1.389059	0.357307
C	2.20972	-0.81808	-1.049227
C	1.506126	0.544362	-1.003041
C	0.180929	0.547458	-0.211314
H	-0.529083	-0.133428	1.728286
H	1.068834	0.589934	1.74436
H	0.417349	-2.181199	0.637765
H	1.252905	-1.823561	2.136692
H	2.87256	-2.379704	0.301162
H	3.109174	-0.74629	0.909556
H	3.172529	-0.710203	-1.557878
H	1.62774	-1.527529	-1.647545
H	1.321769	0.913383	-2.018851
H	2.189463	1.259014	-0.529217
C	-0.870546	-0.254295	-1.032524
H	-0.45306	-1.199623	-1.374442
H	-1.143126	0.337945	-1.910257
C	-2.141104	-0.541648	-0.274305
O	-3.023262	0.250187	-0.033056
C	-0.345994	2.004209	-0.077271
H	-0.47123	2.404347	-1.089824
H	-1.342368	1.98079	0.36767
H	0.488519	2.729371	1.664565
N	0.451024	2.96307	0.680478
H	1.401154	3.031453	0.337106
O	-2.202863	-1.834557	0.144292
H	-3.037562	-1.926787	0.627088

Axial 15			
Atoms	x	y	z
C	-1.345096	-0.045185	1.431327
C	-2.742024	0.264085	0.87923
C	-3.049458	-0.589694	-0.354271
C	-1.96178	-0.426348	-1.419599
C	-0.571504	-0.751294	-0.862961
C	-0.213072	0.078533	0.384182
H	-1.123248	0.60272	2.287917
H	-1.34691	-1.074164	1.81088
H	-2.824884	1.325056	0.62028
H	-3.484506	0.082983	1.662583
H	-3.101512	-1.644285	-0.055486
H	-4.030593	-0.327942	-0.762151
H	-2.167726	-1.080138	-2.272277
H	-1.978725	0.597971	-1.808828
H	0.185579	-0.594775	-1.631668
H	-0.536217	-1.813459	-0.592218
C	1.063809	-0.484839	1.067526
H	1.326333	0.15056	1.917011
H	0.828721	-1.483572	1.442574
C	2.27459	-0.65994	0.18528
O	2.470787	-1.581129	-0.569744
C	3.19205	0.339869	0.35099
H	3.928958	0.127283	-0.240754
H	-0.028104	1.5737	0.039897
H	-0.986148	1.972832	-0.294725
N	0.222529	2.104282	0.970926
H	1.883655	1.876782	-0.665798
O	0.942485	1.810646	-1.03289
H	0.74043	2.679321	-1.511681

Axial 16			
Atoms	x	y	z
C	0.366087	0.037361	1.147633
C	1.808346	-0.385116	1.447904
C	2.211449	-1.580938	0.579542
C	2.032657	-1.267327	-0.908938
C	0.606201	-0.791451	-1.219479
C	0.139108	0.385694	-0.335838
H	-0.29073	-0.796831	1.419612
H	0.062649	0.876977	1.781338
H	1.899862	-0.638555	2.508287
H	2.497682	0.448124	1.268176
H	3.246126	-1.871412	0.784564
H	1.581696	-2.439134	0.845018
H	2.764542	-0.510251	-1.209465
H	2.24992	-2.15575	-1.50928
H	0.529361	-0.498582	-2.273501
H	-0.083649	-1.629214	-1.083747
C	-1.36241	0.678555	-0.601876
H	-1.643895	1.6244	-0.136044
H	-1.515467	0.757829	-1.681436
C	-2.294223	-0.390197	-0.093634
O	-2.487906	-1.468631	-0.606999
C	0.92691	1.66127	-0.750669
H	1.993667	1.435039	-0.75873
H	0.659276	1.895077	-1.786784
H	-0.206059	3.186067	0.068496
N	0.758038	2.876985	0.038872
H	1.069562	2.748087	0.993955
O	-2.917756	-0.018269	1.056159
H	-3.480274	-0.762677	1.316283

Table S04 (Continuation).

Axial 17			
Atoms	x	y	z
C	1.339193	-0.278648	1.406132
C	2.742895	-0.005558	0.850158
C	2.945555	-0.681974	-0.508521
C	1.845676	-0.275099	-1.493254
C	0.45678	-0.594108	-0.92917
C	0.190398	0.075909	0.43069
H	1.268708	-1.346189	1.64827
H	1.193758	0.264575	2.347541
H	2.912674	1.070914	0.749455
H	3.486478	-0.36571	1.567592
H	2.920327	-1.771103	-0.377331
H	3.931784	-0.434609	-0.912176
H	1.97921	-0.795364	-2.446343
H	1.929201	0.796245	-1.710033
H	-0.317657	-0.305861	-1.6468
H	0.365751	-1.680185	-0.807306
C	-1.105623	-0.499539	1.069344
H	-1.336557	0.069759	1.973026
H	-0.921551	-1.538403	1.349684
C	-2.336438	-0.469928	0.199358
O	-3.091621	0.467285	0.05686
C	0.117155	1.628322	0.31614
H	-0.214779	2.013366	1.286233
H	1.129135	2.010716	0.17285
H	-0.410943	1.986056	-1.64306
N	-0.71653	2.237805	-0.711261
H	-1.689447	1.977018	-0.609796
O	-2.539035	-1.651067	-0.436366
H	-3.338669	-1.541868	-0.972203

Equatorial 21			
Atoms	x	y	z
C	0.137477	-1.350703	-1.189748
C	-1.341153	-1.636305	-0.910566
C	-1.544139	-2.031229	0.553859
C	-1.029217	-0.929693	1.482575
C	0.443438	-0.59198	1.208544
C	0.786142	-0.293712	-0.269275
H	0.691911	-2.289729	-1.059349
H	0.277574	-1.049824	-2.233801
H	-1.947702	-0.75831	-1.144496
H	-1.679509	-2.441003	-1.570643
H	-1.001509	-2.963601	0.757783
H	-2.601366	-2.228935	0.75388
H	-1.655319	-0.042473	1.363797
H	-1.129982	-1.234144	2.529117
H	1.04476	-1.459924	1.512927
H	0.754237	0.248435	1.835788
C	0.43517	1.159847	-0.704252
H	1.204325	1.809596	-0.28364
H	0.483737	1.208883	-1.794348
C	-0.904948	1.716762	-0.310657
O	-1.926458	1.665128	-0.95897
O	-0.856412	2.349544	0.893659
H	-1.750136	2.681437	1.063394
C	2.317296	-0.422213	-0.454474
H	2.584429	-1.473419	-0.275821
H	2.56112	-0.201388	-1.498883
H	4.032256	0.579122	0.073478
N	3.071374	0.510959	0.386748
H	3.094122	0.196542	1.350024

Axial 18			
Atoms	x	y	z
C	1.331374	0.22111	-1.425118
C	2.731673	0.445649	-0.839776
C	3.067813	-0.606247	0.220901
C	1.988195	-0.655965	1.305439
C	0.604869	-0.914348	0.697844
C	0.208156	0.128985	-0.364212
H	1.342506	-0.716525	-1.994689
H	1.089651	1.016786	-2.139818
H	3.466154	0.415402	-1.650354
H	2.805028	1.444748	-0.398618
H	4.047226	-0.400028	0.662638
H	3.136627	-1.591319	-0.257581
H	2.216221	-1.443165	2.03008
H	1.988112	0.28722	1.864156
H	0.607717	-1.903279	0.223664
H	-0.154808	-0.97016	1.480813
C	-1.064617	-0.326765	-1.127556
H	-0.837455	-1.272353	-1.626894
H	-1.310994	0.41516	-1.890025
C	-2.284758	-0.592556	-0.28304
O	-2.45592	-1.549657	0.435014
C	0.001175	1.539458	0.251702
H	0.969485	1.924491	0.57562
H	-0.339126	2.209108	-0.54504
H	-1.878856	1.531714	1.100995
N	-0.918751	1.691303	1.37445
H	-0.69361	1.066365	2.138618
O	-3.236109	0.375853	-0.426169
H	-3.989677	0.101215	0.117425

Distorted 1			
Atoms	x	y	z
C	-1.321292	0.838793	0.914538
C	-2.677108	0.758111	0.211151
C	-3.08343	-0.710817	-0.029381
C	-1.849565	-1.609248	-0.259369
C	-0.717004	-0.81744	-0.916847
C	-0.180464	0.297221	0.015175
H	-1.072131	1.862733	1.203201
H	-1.372178	0.254539	1.840923
H	-3.442162	1.27221	0.798839
H	-2.620251	1.291063	-0.743926
H	-3.751315	-0.769198	-0.894169
H	-3.651645	-1.083684	0.828142
H	-1.496313	-2.012628	0.695434
H	-2.117659	-2.473635	-0.872382
H	-1.094837	-0.359141	-1.838977
H	0.096017	-1.482999	-1.2177
C	0.917655	-0.252618	0.965386
H	1.175195	0.535011	1.67603
H	0.520796	-1.107884	1.514212
C	2.187256	-0.656794	0.266716
O	3.057782	0.10089	-0.106388
O	2.273697	-1.996377	0.066823
H	3.110435	-2.152814	-0.395902
C	0.376626	1.437276	-0.862881
H	1.009508	1.001791	-1.648316
H	-0.473839	1.904272	-1.36837
H	2.039243	2.189601	0.054213
N	1.071119	2.463163	-0.080585
H	1.07542	3.345597	-0.577563

Table S04 (Continuation).

Distorted 2			
Atoms	x	y	z
C	1.286085	0.864862	0.916364
C	2.644711	0.875582	0.212216
C	3.140506	-0.564641	-0.033022
C	1.964534	-1.53822	-0.257957
C	0.782725	-0.822586	-0.913867
C	0.173556	0.256175	0.018681
H	1.369862	0.278924	1.838111
H	1.005396	1.876751	1.230147
H	3.375226	1.434451	0.802749
H	2.552616	1.407479	-0.740537
H	3.736702	-0.901145	0.820206
H	3.805441	-0.576559	-0.901523
H	2.283231	-2.383839	-0.871885
H	1.640106	-1.964066	0.696855
H	0.005146	-1.534799	-1.20064
H	1.128329	-0.346132	-1.839598
C	-0.885181	-0.382293	0.954453
H	-1.174102	0.342132	1.721569
H	-0.430708	-1.23662	1.457317
C	-2.136306	-0.895538	0.255048
O	-2.321329	-2.064624	0.007884
C	-0.440565	1.347878	-0.876883
H	0.375754	1.908684	-1.348522
H	-1.011088	0.873502	-1.679747
H	-1.726138	2.954104	-0.758826
N	-1.378736	2.223926	-0.144916
H	-0.911651	2.692411	0.625187
O	-3.041925	0.042706	-0.078713
H	-2.647329	0.935267	0.07997

Distorted 3			
Atoms	x	y	z
C	-0.518458	0.00032	-1.053557
C	-1.972999	0.32766	-1.398376
C	-2.934774	-0.568291	-0.589605
C	-2.338747	-0.940588	0.783963
C	-1.444031	0.186573	1.30119
C	-0.178799	0.366682	0.415691
H	-0.352664	-1.070757	-1.207115
H	0.170887	0.502228	-1.738988
H	-2.146692	0.199527	-2.469619
H	-2.171974	1.383013	-1.181674
H	-3.150891	-1.48089	-1.152079
H	-3.889151	-0.051148	-0.453084
H	-3.13669	-1.153872	1.499439
H	-1.748242	-1.858419	0.699893
H	-1.136574	0.004418	2.33567
H	-2.023416	1.117679	1.310925
C	0.922465	-0.576419	0.976907
H	0.447929	-1.47916	1.362065
H	1.422129	-0.085502	1.820539
C	1.991295	-1.081682	0.008232
O	2.13094	-2.260395	-0.22894
C	0.229112	1.845823	0.543389
H	-0.601566	2.474776	0.202572
H	0.380311	2.063504	1.604472
H	1.295074	2.417703	-1.13325
N	1.482689	2.181644	-0.16393
H	1.916789	2.999124	0.250495
O	2.790056	-0.159859	-0.54613
H	2.447555	0.747836	-0.329188

Table S05. Experimental spectroscopic parameters obtained for the detected rotamers 1-5 of gabapentin using the LA-CP-FTMW technique.

	Rotamer 1	Rotamer 2	Rotamer 3	Rotamer 4	Rotamer 5
A	1272.7056(44)	1486.26558(190)	1217.99250(197)	1361.92329(286)	1311.2210(65)
B	757.67477(117)	652.86193(74)	790.64720(177)	722.24386(65)	736.2311(210)
C	572.88512(63)	546.14752(42)	610.46151(61)	570.32897(52)	570.4553(130)
μ_a	Yes	Yes	Yes	Yes	No
μ_b	Yes	Yes	Yes	Yes	Yes
μ_c	Yes	No	No	No	No
N	63	52	42	60	11
**σ**	86	51	154	25	100

A, *B*, and *C* are the rotational constants measured in MHz. μ_a, μ_b,*and* μ_c represent the three components of the dipole moment, with "Yes/No" indicating if the corresponding transitions has been observed. *N* is the number of measured transitions (only the center of the lines was measured). *σ* is the RMS deviation of the fit (in kHz).

Table S06. Measured frequencies and residuals for the rotational transitions of equatorial 1 conformer in the rotational spectrum obtained with the LA-CP-FTMW.

J'	K'$_{-1}$	K'$_{+1}$	J''	K''$_{-1}$	K''$_{+1}$	ν_{obs}/MHz	$(\nu_{obs}-\nu_{calc})/\ MHz$
**5**	1	5	4	0	4	6176.02750	-0.06785
**6**	2	4	5	3	3	6213.96350	-0.29263
**6**	2	4	5	3	3	6213.96350	-0.29263
**5**	2	4	4	2	3	6561.11910	0.03738
**6**	6	1	6	5	1	6581.87274	-0.17141
**5**	4	1	4	4	0	6750.18126	0.08717
**5**	3	2	4	3	1	6856.23754	0.10111
**5**	1	4	4	1	3	6871.72567	0.12244
**3**	3	1	2	2	0	7010.86005	-0.06363
**3**	3	0	2	2	0	7016.92338	0.00897
**3**	3	1	2	2	1	7052.40205	0.02652
**3**	3	0	2	2	1	7058.36242	-0.00383
**5**	2	3	4	2	2	7086.07945	0.07582
**4**	2	2	3	1	2	7110.71283	0.06836
**6**	1	6	5	1	5	7221.54959	0.00882
**6**	0	6	5	0	5	7252.21588	0.00947
**6**	1	6	5	0	5	7275.30875	-0.04577
**6**	2	5	5	2	4	7803.08408	0.02922
**6**	1	5	5	1	4	8072.53757	0.16469
**6**	3	4	5	3	3	8085.08713	0.05069
**6**	5	1	5	5	0	8090.00543	0.12107
**6**	4	3	5	4	2	8117.12738	0.12880
**6**	4	2	5	4	1	8141.19378	0.08269
**4**	3	2	3	2	1	8255.75266	-0.04645
**4**	3	1	3	2	1	8296.39322	-0.07378
**7**	0	7	6	1	6	8365.53132	-0.04515
**7**	1	7	6	1	6	8375.00444	-0.05332
**7**	0	7	6	0	6	8388.70290	-0.02168
**7**	1	7	6	0	6	8398.22436	0.01850
**4**	3	2	3	2	2	8450.88501	-0.03921
**4**	3	1	3	2	2	8491.53233	-0.05979
**6**	2	4	5	2	3	8510.12760	0.04772
**5**	2	3	4	1	3	8607.64300	0.12885
**7**	2	6	6	2	5	9016.04327	0.01016
**7**	1	6	6	1	5	9208.06453	0.00701
**7**	3	5	6	3	4	9403.24675	0.01954
**8**	0	8	7	1	7	9520.45488	-0.03512
**8**	1	8	7	1	7	9524.24879	0.00758
**8**	0	8	7	0	7	9529.92450	-0.04678
**7**	4	3	6	4	2	9567.67075	0.05372
**4**	4	1	3	3	0	9577.31023	-0.03989
**4**	4	0	3	3	1	9584.00227	-0.05315
**7**	3	4	6	3	3	9859.70273	0.03559
**7**	2	5	6	2	4	9866.53655	0.01790
**5**	3	3	4	2	3	9905.45022	0.04871
**8**	2	7	7	2	6	10204.20830	0.00072
**8**	1	7	7	1	6	10318.83362	-0.09072
**5**	2	3	4	1	4	10414.93798	0.25818
**9**	1	9	8	1	8	10671.34941	-0.02651
**9**	0	9	8	0	8	10673.66305	-0.01779
**8**	4	5	7	4	4	10864.10725	0.02896

**5**	4	1	4	3	1	10903.19257	-0.06449
**5**	4	2	4	3	2	10937.60117	-0.01960
**5**	4	1	4	3	2	10943.89864	-0.02631
**8**	3	5	7	3	4	11359.53171	0.02469
**9**	2	8	8	2	7	11373.97345	-0.02051
**10**	1	10	9	1	9	11817.62920	0.00253
**10**	0	10	9	0	9	11818.51291	-0.01351
**10**	1	10	9	0	9	11819.02996	-0.04301
**19**	4	15	18	6	12	12363.58077	-0.02543
**16**	5	11	16	4	13	12808.94390	-0.07168

Table S07. Measured frequencies and residuals for the rotational transitions of Axial 1 conformer in the rotational spectrum obtained with the LA-CP-FTMW.

J'	K'_{-1}	K'_{+1}	J''	K''_{-1}	K''_{+1}	ν_{obs}/MHz	$(\nu_{obs}-\nu_{calc})/\,MHz$
**5**	2	3	4	2	2	6145.93895	0.00437
**5**	1	5	4	0	4	6160.30744	-0.11969
**6**	1	6	5	1	5	6810.85513	0.04797
**6**	0	6	5	0	5	6919.63362	0.03088
**4**	2	3	3	1	2	7134.83424	-0.11758
**6**	1	6	5	0	5	7148.55620	-0.03151
**6**	2	5	5	2	4	7151.06600	-0.01387
**6**	3	3	5	3	2	7262.31018	-0.02570
**4**	2	2	3	1	2	7274.14082	-0.05223
**6**	1	5	5	1	4	7421.93400	0.05366
**6**	2	4	5	2	3	7424.59978	-0.04217
**7**	0	7	6	1	6	7775.62752	-0.04181
**7**	1	7	6	1	6	7923.34380	0.01737
**7**	0	7	6	0	6	8004.65457	0.00028
**3**	3	1	2	2	0	8027.01056	0.02533
**3**	3	0	2	2	1	8037.08849	-0.04839
**7**	1	7	6	0	6	8152.28779	-0.02361
**7**	2	6	6	2	5	8319.37137	-0.00957
**8**	1	7	7	2	6	8415.74033	0.02742
**7**	4	3	6	4	2	8442.84988	0.06978
**7**	2	5	6	2	4	8704.75838	-0.05750
**8**	0	8	7	1	7	8938.50252	0.04119
**8**	1	8	7	1	7	9030.05242	0.01947
**8**	0	8	7	0	7	9086.12854	0.01011
**8**	1	8	7	0	7	9177.71351	0.0246
**4**	3	2	3	2	1	9205.83321	0.07062
**4**	3	1	3	2	1	9209.61687	0.06869
**4**	3	1	3	2	2	9256.99088	-0.09232
**8**	2	7	7	2	6	9477.64839	-0.04644
**8**	3	6	7	3	5	9650.03634	-0.09088
**8**	1	7	7	1	6	9763.59949	-0.06356
**8**	2	6	7	2	5	9975.47564	-0.08909
**9**	0	9	8	1	8	10077.04911	0.04469
**9**	1	9	8	1	8	10132.18335	0.05720
**9**	1	9	8	0	8	10223.71460	0.01683
**5**	3	3	4	2	2	10348.25424	-0.00116
**5**	3	2	4	2	3	10502.54053	0.03827
**9**	4	5	8	4	4	10894.55666	-0.08191
**4**	4	1	3	3	0	11004.55784	0.00112
**4**	4	0	3	3	1	11005.13390	0.00844
**10**	0	10	9	1	9	11198.42833	0.06037
**10**	0	10	9	0	9	11253.50907	0.01947
**10**	1	10	9	0	9	11285.94569	0.02763
**3**	3	1	2	0	2	11593.22964	-0.01256
**10**	2	9	9	2	8	11762.94727	0.03548
**6**	3	3	5	2	4	11791.09149	-0.02306
**16**	3	14	15	4	12	11813.13165	-0.07219
**5**	4	2	4	3	1	12203.99650	0.02487
**5**	4	1	4	3	2	12208.03733	0.05167
**11**	0	11	10	1	10	12308.41491	0.07992
**6**	4	3	5	3	2	13396.47009	0.02001

Table S08. Measured frequencies and residuals for the rotational transitions of Equatorial 2 conformer in the rotational spectrum obtained with the LA-CP-FTMW.

J'	K'$_{-1}$	K'$_{+1}$	J''	K''$_{-1}$	K''$_{+1}$	ν_{obs}/MHz	$(\nu_{obs}-\nu_{calc})/MHz$
**5**	0	5	4	1	4	6382.51657	0.23420
**5**	1	5	4	0	4	6493.54680	0.06180
**4**	2	3	3	1	2	6613.84249	0.27990
**3**	3	0	2	2	1	6824.17264	0.17871
**6**	0	6	5	1	5	7635.38929	0.09949
**6**	1	6	5	0	5	7681.40277	-0.02177
**4**	3	2	3	2	1	8078.02402	0.09269
**16**	9	7	16	8	8	8118.28802	-0.10536
**6**	2	5	5	1	4	8699.61227	-0.16201
**7**	0	7	6	1	6	8869.77794	0.10055
**7**	1	7	6	0	6	8887.68267	0.06073
**4**	4	1	3	3	0	9229.53919	0.06050
**4**	4	0	3	3	1	9238.31614	0.18022
**5**	3	3	4	2	2	9264.25276	-0.05007
**7**	2	6	6	2	5	9483.14102	-0.03938
**8**	0	8	7	1	7	10095.86777	0.03097
**8**	0	8	7	1	7	10095.87057	0.0338
**8**	1	8	7	1	7	10097.64578	0.02923
**8**	1	8	7	0	7	10102.54066	0.02324
**6**	3	4	5	2	3	10329.77302	-0.22211
**8**	2	6	7	3	5	10602.17461	-0.0289
**8**	2	6	7	3	5	10602.17692	-0.02654
**5**	4	2	4	3	1	10614.84612	-0.00098
**8**	1	7	7	2	6	10675.57692	0.06181
**8**	1	7	7	1	6	10809.89264	0.04849
**8**	2	7	7	1	6	10870.03351	0.01288
**7**	3	5	6	2	4	11302.07727	-0.49244
**9**	0	9	8	1	8	11318.64982	-0.01203
**9**	1	9	8	1	8	11319.27111	-0.02079
**9**	0	9	8	0	8	11320.43168	-0.00992
**9**	1	9	8	0	8	11321.04415	-0.02751
**5**	5	1	4	4	0	11669.67319	0.06458
**5**	5	0	4	4	1	11670.85323	0.07498
**6**	4	3	5	3	2	11929.26766	-0.17816
**8**	3	5	7	3	4	11929.52302	-0.43781
**9**	1	8	8	1	7	12007.87752	0.24325
**9**	2	8	8	1	7	12033.01079	0.09110
**10**	1	10	9	1	9	12540.17200	-0.270
**10**	0	10	9	1	9	12540.17200	-0.05111
**10**	1	10	9	0	9	12541.00691	-0.06491
**10**	0	10	9	0	9	12541.00691	0.15375
**6**	5	2	5	4	1	13079.04298	0.23308

Table S09. Measured frequencies and residuals for the rotational transitions of Equatorial 3 conformer in the rotational spectrum obtained with the LA-CP-FTMW.

J'	K'_{-1}	K'_{+1}	J''	K''_{-1}	K''_{+1}	ν_{obs}/MHz	$(\nu_{obs}-\nu_{calc})/MHz$
**5**	3	2	4	3	1	6571.08752	0.02355
**5**	2	3	4	2	2	6772.88047	0.01024
**7**	2	5	6	3	4	6809.48736	-0.04275
**6**	0	6	5	1	5	7094.81564	0.00031
**3**	3	1	2	2	0	7445.81255	0.00406
**3**	3	0	2	2	1	7472.19260	-0.00457
**6**	3	4	5	3	3	7831.45928	0.02145
**6**	4	3	5	4	2	7834.90099	0.03381
**6**	4	2	5	4	1	7841.85008	0.02886
**6**	3	3	5	3	2	7950.24297	0.029444
**6**	2	4	5	2	3	8173.73132	0.02079
**7**	0	7	6	1	6	8283.03456	0.01797
**7**	1	7	6	0	6	8385.60052	0.01117
**4**	3	2	3	2	1	8687.81021	0.04925
**6**	2	5	5	1	4	8787.98916	-0.00052
**4**	3	1	3	2	2	8820.52245	-0.03158
**7**	2	6	6	2	5	8869.00078	0.00409
**7**	3	5	6	3	4	9130.56669	0.00966
**7**	5	3	6	5	2	9133.91999	0.02001
**7**	1	6	6	1	5	9143.11706	0.06224
**7**	4	4	6	4	3	9159.15143	0.01825
**7**	4	3	6	4	2	9181.55787	0.01104
**7**	3	4	6	3	3	9367.37770	-0.00317
**8**	0	8	7	1	7	9448.88139	0.00967
**8**	1	8	7	0	7	9499.69523	-0.00227
**7**	2	5	6	2	4	9543.29035	0.01611
**7**	2	6	6	1	5	9701.30132	0.00616
**5**	3	3	4	2	2	9848.93263	0.04558
**8**	2	7	7	2	6	10068.76385	-0.0368
**4**	4	1	3	3	0	10182.02874	-0.00698
**4**	4	0	3	3	1	10184.63048	-0.00724
**5**	3	2	4	2	3	10240.04324	-0.04202
**8**	4	5	7	4	4	10486.21119	0.00286
**8**	4	4	7	4	3	10544.57257	-0.00322
**9**	0	9	8	1	8	10602.17461	-0.01312
**9**	1	9	8	1	8	10609.94932	-0.03219
**9**	0	9	8	0	8	10618.80120	0.04929
**9**	1	9	8	0	8	10626.54411	-0.00157
**8**	2	7	7	1	6	10627.08883	0.04781
**8**	3	5	7	3	4	10809.89264	-0.02514
**8**	2	6	7	2	5	10867.81905	-0.00787
**6**	3	4	5	2	3	10907.46664	0.01197
**9**	1	8	8	2	7	11052.87745	-0.01372
**9**	2	8	8	2	7	11250.76671	-0.00275
**9**	1	8	8	1	7	11397.29353	-0.00257
**9**	1	8	8	1	7	11397.29670	0.00061
**5**	4	2	4	3	1	11472.01725	-0.00004
**5**	4	1	4	3	2	11490.37103	0.00112
**10**	0	10	9	1	9	11748.92153	-0.0112
**10**	1	10	9	1	9	11752.49967	-0.02454
**10**	0	10	9	0	9	11756.72298	-0.00357

**10**	1	10	9	0	9	11760.30370	-0.01429
**6**	3	3	5	2	4	11780.61779	0.01200
**7**	3	5	6	2	4	11864.32413	0.02297
**11**	0	11	10	1	10	12892.38495	-0.01755
**11**	1	11	10	1	10	12893.98277	-0.04751
**11**	0	11	10	0	10	12896.01139	0.01744
**11**	1	11	10	0	10	12897.57188	-0.04985
**5**	5	1	4	4	0	12907.16446	-0.05288
**5**	5	0	4	4	1	12907.41298	-0.01844

Table S10. Measured frequencies and residuals for the rotational transitions of Equatorial 4 conformer in the rotational spectrum obtained with the LA-CP-FTMW.

J'	K'_{-1}	K'_{+1}	J''	K''_{-1}	K''_{+1}	ν_{obs}/MHz	$(\nu_{obs}-\nu_{calc})/MHz$
**3**	3	1	2	2	0	7196.46618	0.09002
**6**	1	6	5	0	5	7263.59770	-0.14846
**7**	0	7	6	1	6	8309.23591	0.07406
**7**	1	7	6	0	6	8370.19142	-0.03437
**4**	3	2	3	2	1	8438.18574	-0.02202
**4**	3	1	3	2	2	8611.75856	-0.01354
**6**	2	5	5	1	4	8612.21806	-0.02615
**5**	3	3	4	2	2	9582.18343	-0.20331
**4**	4	1	3	3	0	9834.76088	0.03908
**4**	4	0	3	3	1	9838.82513	0.04898
**8**	2	7	7	1	6	10487.35266	0.16329

Table S11. Measured frequencies and residuals for the rotational transitions of Equatorial 1 conformer in the rotational spectrum obtained with the LA-MB-FTMW.

J'	*K'-1*	*K'+1*	*F'*	*J"*	*K"-1*	*K"+1*	*F"*	ν_{obs}/MHz	$(\nu_{obs} - \nu_{calc})/MHz$
**4**	1	4	3	3	0	3	2	5109.18865	0.00272
**4**	1	4	5	3	0	3	4	5109.23865	-0.00172
**5**	1	5	4	4	0	4	3	6176.02305	-0.00679
**5**	1	5	6	4	0	4	5	6176.04265	0.00292
**5**	1	5	5	4	0	4	4	6176.26315	-0.00173
**6**	1	6	7	5	0	5	6	7275.32300	0.00137
**6**	1	6	6	5	0	5	5	7275.46020	0.00459
**7**	1	7	8	6	0	6	7	8398.18595	-0.00074
**7**	1	7	6	6	0	6	5	8398.20095	0.00352
**7**	1	7	7	6	0	6	6	8398.27095	-0.00103
**8**	1	8	9	7	1	7	8	9524.22873	-0.00074
**8**	1	8	8	7	1	7	7	9524.27756	-0.00227

Table S12. Measured frequencies and residuals for the rotational transitions of Axial 1 conformer in the rotational spectrum obtained with the LA-MB-FTMW.

J'	*K'-1*	*K'+1*	*F'*	*J"*	*K"-1*	*K"+1*	*F"*	ν_{obs}/MHz	$(\nu_{obs} - \nu_{calc})/MHz$
**3**	1	3	2	2	0	2	1	4167.10460	-0.00028
**3**	1	3	4	2	0	2	3	4167.29570	0.00031
**4**	1	4	5	3	0	3	4	5173.10615	-0.00099
**4**	1	4	4	3	0	3	3	5173.82442	0.00338
**5**	1	5	6	4	0	4	5	6160.32550	-0.00388
**5**	1	5	5	4	0	4	4	6160.94780	-0.00261
**6**	1	6	5	5	0	5	4	7148.49730	0.00567
**6**	1	6	7	5	0	5	6	7148.53650	0.00186
**6**	1	6	6	5	0	5	5	7149.01180	-0.00089
**7**	1	7	6	6	0	6	5	8152.27595	-0.00205
**7**	1	7	8	6	0	6	7	8152.29595	-0.00008
**7**	1	7	7	6	0	6	6	8152.64095	-0.00030

Table S13. Measured frequencies and residuals for the rotational transitions of Equatorial 2 conformer in the rotational spectrum obtained with the LA-MB-FTMW.

J'	*K'₋₁*	*K'₊₁*	*F'*	*J"*	*K"₋₁*	*K"₊₁*	*F"*	ν_{obs}/MHz	$(\nu_{obs}-\nu_{calc})/MHz$
**3**	1	3	3	2	0	2	2	4200.02441	-0.00114
**3**	1	3	4	2	0	2	3	4200.55370	-0.00038
**4**	1	4	4	3	0	3	3	5334.57985	-0.00254
**4**	1	4	5	3	0	3	4	5334.91485	0.00176
**4**	1	4	3	3	0	3	2	5334.97985	-0.00226
**5**	1	5	5	4	0	4	4	6493.30620	0.00591
**5**	1	5	6	4	0	4	5	6493.48750	0.00278
**6**	1	6	6	5	0	5	5	7681.34215	0.00200
**6**	1	6	7	5	0	5	6	7681.44505	0.00045
**7**	0	7	7	6	1	6	6	8869.73285	0.00545
**7**	0	7	6	6	1	6	5	8869.75535	-0.00577
**7**	0	7	8	6	1	6	7	8869.77285	0.00257
**7**	1	7	7	6	0	6	6	8887.58285	-0.00383
**7**	1	7	8	6	0	6	7	8887.64785	-0.00433

Table S14. Measured frequencies and residuals for the rotational transitions of Equatorial 3 conformer in the rotational spectrum obtained with the LA-MB-FTMW.

J'	*K'₋₁*	*K'₊₁*	*F'*	*J"*	*K"₋₁*	*K"₊₁*	*F"*	ν_{obs}/MHz	$(\nu_{obs}-\nu_{calc})/MHz$
**5**	1	5	4	4	0	4	3	6227.50738	-0.00112
**5**	1	5	5	4	0	4	4	6227.44146	-0.00047
**5**	1	5	6	4	0	4	5	6227.50245	-0.00070
**6**	1	6	7	5	0	5	6	7292.49360	0.00140
**6**	1	6	6	5	0	5	5	7292.45440	-0.00172
**8**	1	8	9	7	0	7	8	9499.71385	0.00451
**8**	1	8	7	7	0	7	6	9499.71385	0.00317
**7**	1	7	8	6	0	6	7	8385.61595	0.00126
**7**	1	7	7	6	0	6	6	8385.59595	0.00577
**4**	1	4	5	3	0	3	4	5187.03080	0.00158
**4**	1	4	4	3	0	3	3	5186.94015	-0.00131
**4**	1	4	3	3	0	3	2	5187.04550	0.00029
